# Effect of the Rate of Glucose Consumption on the Total Peroxyl Radical Trapping Antioxidant Potential (TRAP) of Plasma in Overweight Men and Women: A Randomized Trial

**DOI:** 10.3390/antiox15040512

**Published:** 2026-04-21

**Authors:** Shannan M. Grant, Thomas M. S. Wolever, Alexandra Thompson, Laura Chiavaroli, Maxine Seider, Antonia Harvey, Megan Gray, Pauline Darling, Deborah O’Connor, Robert G. Josse, Kazimiera A. Mizier-Barre, David Kitts, Douglas Edward Barre

**Affiliations:** 1 Department of Nutritional Sciences, Faculty of Medicine, University of Toronto, Toronto, ON M5S 1A8, Canada; shannan.grant2@msvu.ca (S.M.G.); laura.chiavaroli@utoronto.ca (L.C.); maxine.seider@sunnybrook.ca (M.S.); deborah.oconnor@utoronto.ca (D.O.); robert.josse@utoronto.ca (R.G.J.); 2 Department of Applied Human Nutrition, Faculty of Professional Studies, Mount Saint Vincent University, Halifax, NS B3M 2J6, Canadamegan.gray@dal.ca (M.G.); 3 Department of Obstetrics and Gynecology, Faculty of Medicine, Dalhousie University, Halifax, NS B3H 4R2, Canada; 4 Department of Obstetrics and Gynecology, IWK Health, Halifax, NS B3K 6R8, Canada; 5 Toronto 3D Knowledge Synthesis and Clinical Trials Unit, Clinical Nutrition and Risk Factor Modification Centre, St. Michael’s Hospital, Toronto, ON M5C 2T2, Canada; 6 Li Ka Shing Knowledge Institute, St. Michael’s Hospital, Toronto, ON M5C 2T2, Canada; 7 School of Nutrition Sciences, Faculty of Health Sciences, University of Ottawa, Ottawa, ON K1N 6N5, Canada; paulinedarling@gmail.com; 8 Department of Biology, School of Science and Technology, Cape Breton University, Sydney, NS B1P 6L2, Canada; 9 Department of Food Science, Faculty of Land and Food Systems, University of British Columbia, Vancouver, BC V6T 1Z4, Canada; david.kitts@ubc.ca; 10 Department of Health Sciences, School of Education and Health, Cape Breton University, Sydney, NS B1P 6L2, Canada; ed_barre@cbu.ca

**Keywords:** glucose, glycemic index, carbohydrate, randomized clinical trial, humans, sipping, oxidized LDL, conjugated dienes

## Abstract

Low glycemic-index foods may reduce postprandial oxidative stress by reducing postprandial glucose excursions, but the evidence for this is limited by dietary confounders. To determine whether reducing postprandial glucose per se reduces postprandial oxidative stress, overnight-fasted participants (BMI 25.0–39.9 kg/m^2^, n = 18) consumed four test meals in random order: 75 g dextrose solution (Dex) within 5 min (bolus/noC), Dex slowly over 3.25 h (sipping/noC), bolus with 1 g vitamin C (bolus/C) and sipping with 1 g vitamin C (sipping/C). Venous blood was taken at intervals over 6 h; a standard lunch was consumed at 4 h. Sipping flattened postprandial glucose and insulin and reduced free fatty acid rebound compared to bolus (*p* < 0.05). Vitamin C raised serum vitamin C from ~20 to ~55 μmol/L. The total peroxyl radical trapping antioxidant potential (TRAP) increments differed after lunch, with a main effect of vitamin C at 5 h (mean ± SEM; C 70 ± 23 vs. noC −29 ± 27; *p* = 0.016) and main effects of rate (sipping 57 ± 25 vs. bolus −71 ± 28; *p* = 0.0002) and vitamin C (C 58 ± 25 vs. noC −73 ± 28; *p* = 0.0003) at 6 h. By multiple regression analysis, the TRAP area under the curve (AUC) was positively associated with the insulin AUC (*p* < 0.001) and negatively with the glucose and vitamin C AUCs (*p* < 0.05). The oxidized LDL increments were higher 6 h after sipping than bolus (7 ± 7 vs. −20 ± 7, *p* = 0.005). The oxidized LDL AUC was negatively associated with the TRAP AUC (*p* < 0.001). These results support the hypothesis that reducing postprandial glucose reduces postprandial oxidative stress.

## 1. Introduction

The glycemic index (GI) quantifies the ability of the available carbohydrate (avCHO) in moderate- to high-carbohydrate foods to raise postprandial blood glucose relative to the same amount of avCHO from glucose [1,2,3]. Treatment of type 2 diabetes with low-GI diets elicits small but statistically and clinically relevant reductions in glycated hemoglobin, serum cholesterol and body weight [4]. In people without diabetes, low-GI diets are associated with significantly reduced risks of developing type 2 diabetes, cardiovascular disease, diabetes-related cancers, and total mortality [5,6]. These effects may be due, in part, to a reduction in oxidative stress elicited by low-GI foods. Low-GI starchy foods are digested in vitro at a slower rate than high-GI foods [7,8,9]. Reducing the rate of carbohydrate absorption leads to a flatter glycemic response characterized by a lower peak rise and slower return of blood glucose to the fasting concentration [10].

Spikes of postprandial glucose elicit postprandial oxidative stress and endothelial dysfunction [11,12], effects that may contribute to the pathogenesis of diabetes [13], diabetes complications [14] and cardiovascular disease [15]. It has been hypothesized that low-GI test meals [16] and diets [17] reduce postprandial oxidative stress by reducing postprandial glucose spikes. However, the results of these studies may be confounded by differences in the antioxidant content of the test meals and diets. Therefore, retesting the hypothesis that reducing glucose excursions per se reduces postprandial oxidative stress is warranted. We hypothesized that postprandial glucose spikes could be reduced in the absence of confounding dietary factors by consuming 75 g dextrose slowly (sipping) as compared to rapidly (bolus) [10]. Therefore, we used this model to determine the effect of reducing postprandial glucose spikes per se on the primary endpoint of postprandial total peroxyl radical trapping antioxidant potential (TRAP). TRAP is considered to be a useful tool to assess the overall antioxidant capacity of human serum [18]. The sipping and bolus treatments were administered with and without 1 g of vitamin C to determine if the effect of reducing the rate of dextrose intake was similar to that of adding vitamin C.

## 2. Materials and Methods

This study had a single-blind randomized cross-over (2 × 2 factorial) design (technicians running the biochemical analyses were blinded to treatment). The clinical trial portion of this study was conducted at INQUIS Clinical Research (formerly GI Labs), Toronto, Ontario. The first study visit occurred on 24 August 2010 and the last visit on 10 July 2011. The trial was posted on clinicaltrials.gov as NCT01440790 on 23 August 2011; although this was after the last subject visit, it was before the results of the primary outcome were available. Study participants were recruited from GI Labs and the Department of Nutritional Sciences, University of Toronto, using posters and snowball/referral sampling.

The study was conducted in accordance with the Declaration of Helsinki, and the protocol was reviewed and approved by the University of Toronto Research Ethics Board on 6 August 2010 (protocol reference #25401). Informed consent for participation was obtained from all subjects involved in the study. Individuals interested in participating had the study procedures explained to them and were invited to review the study consent form. Participants were encouraged to ask any questions they had and not to sign the consent form until all their questions were answered to their satisfaction.

### 2.1. Screening

After providing informed consent, participants completed a researcher-administered questionnaire (screening questionnaire) and gave a blood sample to ensure all eligibility criteria were met. Participants were men or women aged 18–65 with a BMI between 25.0 and 39.9 kg/m^2^, inclusive, who were willing and able to comply with the protocol. Exclusion criteria were one or more of: fasting glucose ≥ 7 mmol/L, serum creatinine > 1.7 times the upper limit of normal (ULN), aspartate transaminase and/or alanine transaminase > 2 times the ULN, unstable cardiovascular disease, thyroid stimulating hormone outside the normal limits, serum triglycerides > 4.0 mmol/L, low-density lipoprotein (LDL) cholesterol > 5.0 mmol/L, systolic blood pressure (SBP) > 160, diastolic blood pressure (DBP) > 100 mg/Hg, cigarette smokers, high alcohol intake (females, >2 drinks/d or >10/wk; males, >3 drinks/d or >15/wk), regular consumption of antioxidant supplements, weight loss of ≥10 lbs or major changes to physical activity in the month prior to screening.

### 2.2. Procedures

On four separate occasions, after a 12 h overnight fast, participants attended a 6.5-h (390 min) study visit. After participants arrived, they were weighed and had height and waist circumference measured; a cannula was then inserted into a forearm vein in the left arm and a fasting blood sample obtained. To facilitate blood taking, participants’ arms were kept warm with heating pads (set on low heat) between blood samples. After each blood sample the cannula was flushed with a small volume of saline to prevent it being blocked by a blood clot. Prior to each subsequent blood sample 2–3 mL of fluid was withdrawn and discarded to clear the saline from the line.

After the fasting blood sample had been obtained, one of the 4 study treatments was administered in random order (by S.M.G. and L.C.). The order of treatments was created using the “@rand” command in Lotus 1-2-3 1997 edition (Lotus Development Corporation, Cambridge, MA, USA) by T.M.S.W. and assigned to participants in the order they consented. The four treatments were: (1) 75 g dextrose dissolved in 250 mL of tap water consumed within 5 min (bolus/noC); (2) 75 g dextrose dissolved in 250 mL of tap water consumed over 3.25 h at a rate of ~77 mL/h (sipping/noC); (3) bolus plus 1 g oral vitamin C (bolusC); and (4) sipping plus 1 g oral vitamin C (sippingC). Treatments were administered in eight fluid ounce glasses. For sipping treatments, the glass was measured and marked with black marker to indicate the volume to be consumed at each sip over the consumption period. A timer was started at the first sip and additional blood samples collected at 30, 60, 120, 180, 240, 270, 300 and 360 min; additional measurements of cardiovascular hemodynamics were obtained after the 60, 120, 240, 300 and 360 min blood samples. After the 240 min blood sample, participants consumed a standard lunch within 25 min consisting of 2 slices (70 g) whole wheat bread, 2 tablespoons (28 g) mayonnaise, 1.5 leaves of iceberg lettuce (10–12 g), 1.3 cup peeled cucumber (40 g), 240 mL of 2% milk, one Black Dimond cheese string (21 g) and one medium apple with peel (137 g). The lunch meal contained 655 kcal with 64 g (39% of energy) carbohydrate, 35 g (48% of energy) fat, 21 g (13% of energy) protein and 8.5 mg vitamin C. After obtaining the last blood sample and last measurements of blood pressure and augmentation index (AI), the cannula was removed, and participants were offered a light snack and observed for signs and symptoms for 20–30 min prior to leaving the clinic.

Blood samples were drawn into three different tubes (Vacutainer^®^ Blood Collection Tubes, BD-Canada, Mississauga, ON, Canada). Table 1 shows the container types, volumes, and times at which blood samples for the various outcomes were obtained. Gold-top tubes were spun at 3000 rpm at 4 °C for 15 min and the serum transferred to labeled microtubes. Lavender-top tubes were spun at 2500 rpm at 4 °C for 15 min and green-top at 2500 rpm at room temperature for 10 min and the plasma from both types transferred into labeled amber (photo-protective) microtubes. All serum/plasma microtubes were stored at −80 °C prior to analysis.

Participant weight (kg) and height (m) were measured using a calibrated SECA Medical Beam Scale, and BMI calculated as kg/m^2^. Waist circumference was measured by trained clinical staff (S.M.G., A.T., or L.C.). Anthropometric data were collected in duplicate at the screening appointment and each study visit; average values were used in analysis. Participants were asked to avoid intentionally changing their body weight during the study period and reminded during each phone call to book study appointments.

The following traditional and non-traditional markers of cardiovascular risk, referred to as “Cardiovascular Hemodynamics Outcomes” or “Vitals”, were collected during the study: diastolic blood pressure (DBP, mmHg), systolic blood pressure (SBP, mmHg), pulse pressure (PP = SBP − DBP), augmentation index (AugIdx), AugIdx normalized to a PP of 75 mmHg (AIp75), heart rate (beats per minute; BPM). The Omeron Non-invasive Blood Pressure Monitor with AugIdx (HEM-9000AI, Omron Healthcare Inc., Bannockburn, IL, USA) was used to measure participants’ blood pressure (BP) and calculate their AugIdx. Adhering to in-lab standard operating procedures based on Jovanovski et al. [19], the HEM-9000AI Instruction Manual and Van Bortel et al. [20], BP was obtained via the digital oscillometric method using a BP cuff [19,20]. The AI calculation was based on several components of a participant’s pulse wave, which was obtained via applanation tonometry using a sensor placed against the radial artery. A supplementary transfer function was not applied to the data collected for this study [21,22,23]. Vital measurements were conducted in a quiet temperature-controlled room by one of two trained clinical scientists at baseline, 60, 120, 240, 300, and 360 min. For each measurement of vitals, participants were seated in an upright position so that they could rest their elbow comfortably on a table set to height just below their sternum. After a 15-min resting period, DBP and SBP were measured in duplicate from the left arm. Immediately after blood pressure had been measured, AugIdx was measured in duplicate. The location used to measure AugIdx at baseline was marked with black marker and the same location used for each subsequent measurement. The average value of duplicate measurements was recorded. Raw and normalized values for AugIdx and AIp75 were provided by the device. AugIdx measurements were not collected for one participant because of inadequate vessel compression.

### 2.3. Analytical Methods

TRAP, oxidized low-density lipoprotein cholesterol (oxLDL), and conjugated dienes per Apolipoprotein B-100 (CD/ApoB-100) were analyzed in the Department of Health Sciences and Emergency Management at Cape Breton University (S.M.G., D.E.B., K.A.M.-B.). The TRAP assay was based on the spectrophotometric assay developed by Valkonen and Kuusi [24] to measure TRAP in ethylenediaminetetraacetic acid (EDTA) plasma. For this study, this assay was adapted for analysis using a microplate reader (Spectramax 190, Molecular Devices, Sunnyvale, CA, USA). Coefficients of variation (CVs) were measured for duplicate lag times of each of plasma and Trolox. A CV less than 10% was acceptable. If the CV was not met, samples were rerun. OxLDL was analyzed using the Mercodia Oxidized LDL ELISA kit (Mercodia AB, Sylveniusgatan, BA, Sweden) modified as follows: (1) for the first dilution, 15 μL of plasma was mixed with 1200 μL of sample buffer; (2) for the second dilution, 15 μL of the initial dilution was mixed with 1200 μL of sample buffer for a final dilution of 1/6400. If the oxLDL values produced by the microplate reader were beyond the range of the standard curve, the plasma was diluted to 1:4 or 1:6 (10 μL of plasma plus 30 or 50 μL of ultra-high-purity water). The concentrations of conjugated dienes (CD) and protein in LDL particles were measured after heparin citrate precipitation of LDL particles, as described by Ahotupa et al. [25,26] for CD and Petersen [27] for protein. For this study, these assays were adapted for analysis using a microplate reader (Spectramax 190, Molecular Devices, Sunnyvale, CA, USA). Since the only protein in LDL particles is ApoB-100, and, since there is one molecule of ApoB-100 per LDL particle, the amount of CD per LDL particle, is indicated by the CD/ApoB-100 concentration ratio.

Vitamin C was analyzed at Hospital in Common Laboratory (London Health Sciences Centre, London, ON, Canada) by high-pressure liquid chromatography (HPLC) according to the method of Wagner et al. [28].

Glucose and insulin were analyzed at INQUIS. Glucose was measured using a Model 2300 STAT glucose analyzer (Yellow Springs Instruments, Yellow Springs, OH, USA); analytical CV < 2%. Insulin was measured by enzyme-linked immunosorbent assay (ELISA) (ALPCO Insulin ELISA; Salem, NH, USA); analytical CV 10.3%. Each fasting glucose and insulin sample was measured twice, and the mean was used as the fasting value.

Free fatty acids (FFAs) were measured at the J. Alick Little Lipid Research Laboratory at St. Michael’s Hospital, Toronto, using the non-esterified fatty acids, NEFA-HR (2) Assay (Wako Diagnostics, Richmond, VA, USA).

### 2.4. Calculations

For each endpoint, increments were calculated by subtracting the fasting value from each postprandial value. For glucose and insulin, incremental areas under the curve (iAUCs), ignoring the area beneath the fasting value, were calculated as described elsewhere [29]; using this method, iAUC is always ≥0. For all other endpoints net incremental area under the curve (netAUC) was calculated by applying the trapezoid rule to the positive and negative increments; using this method, netAUC can be <0. Values for iAUC and netAUC were calculated using the fasting value as baseline from 0 to 4 h (iAUC04 and netAUC04), 4 to 6 h (iAUC46 and netAUC46) and 0 to 6 h (iAUC06 and netAUC06). In addition, iAUC and netAUC from 4 to 6 h were calculated using the value at 4 h as the baseline (iAUC4b6 and netAUC4b6). The maximum amplitude of the excursion (MAE) was calculated as maximum concentration achieved minus the minimum concentration achieved.

### 2.5. Statistical Analysis

Data for each endpoint were submitted to repeated-measures analysis of variance (ANOVA) using the linear model to examine the main effects of time, rate (sipping vs. bolus, i.e., the mean of [sipping/noC and sipping/C] vs. the mean of [bolus/noC and bolus/C]) and vitamin C (VitC or no VitC, i.e., the mean of [sipping/C and bolus/C] vs. the mean of [sipping/noC and bolus/noC]) and the time × rate, time × VitC, rate × VitC, and time × rate × VitC interactions. If there were significant main effects (*p* ≤ 0.05) or interactions (*p* ≤ 0.10), values at each time point and each AUC and MAE were subjected to ANOVA to examine the main effects of rate and vitamin C and the rate × VitC interaction; if the interaction was significant (*p* < 0.05), then the significance of the differences between individual means was determined by Tukey’s test to adjust for multiple comparisons, with the criterion for significance being 2-tailed *p* < 0.05. If there were no significant main effects (*p* > 0.05) or interactions (*p* > 0.10), then the results of the additional statistical tests were not considered to be conclusive.

The associations of the AUCs for serum glucose, insulin, FFA and plasma vitamin C AUCs (AUC04, AUC06, and AUC4b6; independent variables) and the respective AUCs for TRAP and the associations among the AUCs of glucose, insulin, FFA, vitamin C and TRAP on oxLDL, CD/ApoB-100 and CRP were assessed by multiple regression analysis using the step-up method. For each multiple regression analysis, the residuals were calculated and outliers (defined as residuals whose absolute value was greater than 2× SD of all the residuals) identified. The results shown are those based on a second regression analysis carried out after excluding the outliers.

Artificial intelligence (GenAI) was not used for any purpose during protocol development, implementation, analysis, or for the writing of this paper.

## 3. Results

Twenty-eight (28) participants expressed interest in the study, 24 were screened, and 18 were eligible (Supplementary Figure S1); they comprised seven males and 11 females, respectively, age (mean ± SD) 51 ± 8 and 51 ± 7 yr, BMI 33 ± 3 and 33 ± 6 kg/m^2^, waist circumference 96 ± 12 and 99 ± 13 cm, and blood pressure 138 ± 10/79 ± 10 and 127 ± 14/78 ± 10. Of the 18 participants who started the study, one dropped out after the first visit due to dizziness/nausea after consuming the bolus treatment, while another subject dropped out due to excessive bruising at the catheter site. The remaining 16 participants successfully completed all four visits.

### 3.1. Fasting Concentrations

The fasting concentrations prior to the consumption of each treatment did not differ significantly among treatments for any of the 18 endpoints (Supplementary Table S1).

### 3.2. Glucose, Insulin, FFA and Vitamin C

**Figure 1:**
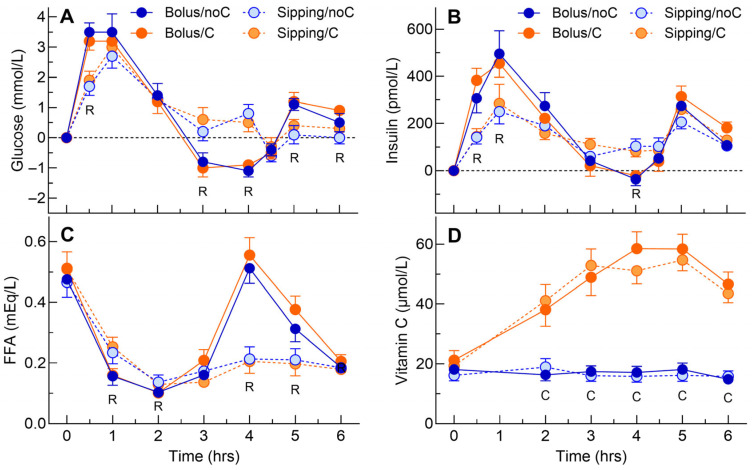
Glucose, insulin, FFA and vitamin C increments. Values are means ± SEM for n = 16 subjects after consuming 75 g dextrose within 5 min without (bolus/noC, dark blue) or with 1 g vitamin C (bolus/C, dark orange) or 75 g dextrose sipped evenly over 3.25 h without (sipping/noC, light blue) or with 1 g vitamin C (sipping/C, light orange). Panels: (**A**) plasma glucose increments (mmol/L); (**B**) serum insulin increments (pmol/L); (**C**) serum free fatty acid concentrations (mEq/L); (**D**) serum vitamin C concentrations (μmol/L). R = significant main effect of rate (*p* < 0.05). C = significant main effect of vitamin C (*p* < 0.05).

The main effect of time and the time × rate interaction were significant for both plasma glucose concentrations and increments (Supplementary Table S2). Sipping flattened the glucose response curve, with the increments for sipping being significantly less than bolus at 0.5, 5, and 6 h and significantly above bolus at 3 and 4 h (Figure 1A). Neither rate nor vitamin C significantly affected glucose iAUC04 or iAUC06; however, iAUC4b6 and MAE were both significantly lower after sipping vs. bolus (Table 2).

The main effects of time and rate and the time × rate interaction were significant for insulin concentrations and increments (Supplementary Table S2). Sipping flattened the insulin response curve, with the increments for sipping being significantly less than bolus at 0.5 and 1 h and significantly above bolus at 4 h (Figure 1B). Neither rate nor vitamin C significantly affected insulin iAUC04 or iAUC06; however, iAUC4b6 and MAE were both significantly lower after sipping vs. bolus (Table 2).

The main effects of time and rate and the time × rate interaction were significant for FFA concentrations and increments (Supplementary Table S2). After sipping, the mean serum FFA concentrations were significantly higher than after bolus at 0.5 and 1 h but less than bolus at 4 and 5 h (Figure 1C). Neither rate of glucose intake nor presence of vitamin C significantly affected FFAs nAUC04 or nAUC06; however, nAUC4b6 and MAE were significantly lower after sipping than bolus (Table 2).

The main effects of time and vitamin C and the time × VitC interaction were significant for serum vitamin C concentrations and increments (Supplementary Table S2). The serum vitamin C concentrations were significantly higher from 2 to 6 h after vitamin C compared to no vitamin C (Figure 1D). Vitamin C significantly increased serum vitamin C nAUC04, nAUC06 and MAE but had no significant effect on nAUC4b6 (Table 2).

### 3.3. TRAP (Primary Endpoint)

**Figure 2:**
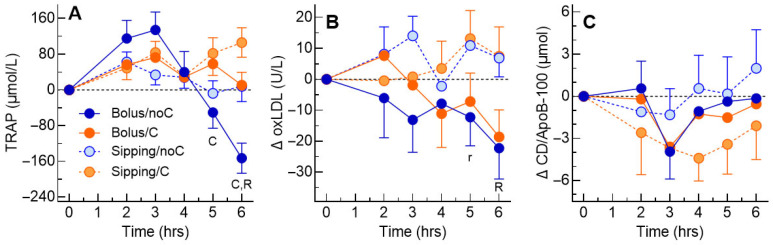
TRAP, oxidized LDL and conjugated dienes. Values are means ± SEM for n = 16 subjects after consuming 75 g dextrose within 5 min (bolus, dark blue circles) or 75 g dextrose evenly over 3.25 h (sipping, light blue circles), or bolus plus 1 g vitamin C (bolus + C, dark orange circles) or sipping plus 1 g vitamin C (sipping + C, light orange circles). Panels: (**A**) increments of serum total peroxyl radical trapping antioxidant potential (TRAP, µmol/L); (**B**) increments of serum oxidized LDL (oxLDL, U/L); (**C**) increments of serum conjugated dienes per apoB100 (μmol/μmol). R = significant main effect of rate (*p* < 0.05); r = main effect of rate (*p* = 0.0500); C = significant main effect of vitamin C (*p* < 0.05).

The main effect of time and the time × VitC interaction were significant for TRAP concentrations, with time, time × rate and time × VitC being significant for TRAP increments (Supplementary Table S2). The mean TRAP increments after the four treatments were similar to each other from 2 to 4 h; however, at 5 h, they were higher after VitC than no VitC (70 ± 23 vs. −29 ± 27, *p* = 0.004), and, at 6 h, they were higher after sipping than bolus (57 ± 25 vs. −71 ± 21, *p* = 0.0002) and higher after VitC than no VitC (58 ± 25 vs. −73 ± 28, *p* = 0.003) (Figure 2A). At 6 h, the mean TRAP increment after bolus/noC was significantly lower than those for all the other treatments. Neither rate nor vitamin C significantly affected TRAP nAUC04 or nAUC06; however, nAUC4b6 was significantly higher after sipping than bolus and higher after VitC than no VitC (Table 3). There was a significant rate × vitC interaction for MAE, with the mean after bolus/noC being greater than all the other treatments (Table 3).

**Table 3:** AUCs and maximum excursions for measures of oxidative stress.

Endpoint	Measure	Individual Treatments				Means for Main Effects			
		Bol-C (B)	Bol+C (BC)	Sip-C (S)	Sip+C (SC)	Bolus (B+BC)	Sipping (S+SC)	No VitC (B+S)	VitC (BC+SC)
TRAP ^3^ (primary endpoint)	nAUC04 ^1^	326 ± 116	171 ± 71	141 ± 50	172 ± 66	249 ± 69	157 ± 42	234 ± 67	172 ± 57
	nAUC06 ^1^	219 ± 160	249 ± 92	151 ± 92	323 ± 119	234 ± 96	237 ± 84	185 ± 113	286 ± 93
	nAUC4b6 ^2^	−187 ± 54	21 ± 44	−46 ± 22	88 ± 29	−83 ± 40	21 ± 19 *	−117 ± 32	54 ± 29 *
	MAE	350 ± 52 ^a^	212 ± 26 ^b^	190 ± 20 ^b^	214 ± 23 ^b^	281 ± 27	202 ± 18	270 ± 30	213 ± 20
oxLDL ^3^	nAUC04 ^1^	−26 ± 32	4 ± 23	25 ± 20	2 ± 29	−11 ± 18	13 ± 16	−1 ± 20	3 ± 14
	nAUC06 1	−54 ± 48	−18 ± 38	38 ± 31	20 ± 45	−36 ± 29	29 ± 27	−8 ± 31	1 ± 23
	nAUC4b6 ^1^	−12 ± 15	0 ± 13	18 ± 13	12 ± 10	−6 ± 10	15 ± 9	3 ± 6	6 ± 6
	MAE ^1^	68 ± 9	55 ± 9	53 ± 9	54 ± 7	61 ± 8	53 ± 7	60 ± 8	54 ± 6
CD/apoB ^3^	nAUC04 ^1^	−3.6 ± 4.9	−4.5 ± 4.6	−2.7 ± 4.9	−9.8 ± 5.9	−4.1 ± 3.1	−6.3 ± 4.3	−3.2 ± 3.6	−7.2 ± 3.9
	nAUC06 ^1^	−4.6 ± 8.2	−7.0 ± 6.8	−1.3 ± 8.7	−16.5 ± 9.4	−5.8 ± 4.2	−8.9 ± 7.1	−3.0 ± 6.6	−11.7 ± 5.7
	nAUC4b6 ^1^	1.2 ± 2.3	0.1 ± 2.3	0.3 ± 4.4	2.1 ± 1.9	0.6 ± 2.0	1.2 ± 2.4	0.8 ± 2.4	1.1 ± 1.8
	MAE ^1^	14.0 ± 2.1	11.5 ± 1.4	14.2 ± 2.6	13.6 ± 1.7	12.8 ± 1.4	13.9 ± 1.5	14.1 ± 1.8	12.6 ± 1.1

Values are means ± SEM for n = 16 subjects. TRAP = total peroxyl radical trapping antioxidant potential, oxLDL = oxidized low-density lipoprotein cholesterol; CD/apoB = conjugated dienes/apolipoprotein B100 (μmol/μmol); nAUC = net incremental area under the curve (negative area below baseline included); nAUC04 and nAUC06 are the nAUC calculated from 0 to 4 h and 0 to 6 h, respectively, using the fasting (0 min) value as the baseline; nAUC4b6 is the AUC calculated from 4 to 6 h using the value at 4 h (before lunch) as the baseline. MAE is the maximum amplitude of the excursion (maximum minus minimum concentration from 0 to 6 h). * Significant main effect: *p* < 0.05 (orange); *p* < 0.001 (green). ^ab^ Significant rate × VitC interaction: means with different letter superscripts differ significantly by Tukey’s test (*p* < 0.05; orange). ^1^ No significant main effects and no significant rate × vitC interaction. ^2^ No significant rate × vitC interaction. ^3^ Units: TRAP; nAUC, units·min/L; MAE, units/L. oxLDL; nAUC, units·min/L; MAE, units/L. CD/apoB; nAUC, μmol·min/μmol; MAE, μmol/μmol.

### 3.4. oxLDL and CD/ApoB-100

There was no significant main effect of time or VitC and no significant interactions for oxLDL concentrations or increments (Supplementary Table S2). There were no significant differences in the AUCs or MAE (Table 3), but the mean oxLDL increment at 6 h was lower after bolus than sipping (*p* = 0.005, Figure 2B). However, this effect may not be conclusive since the *p*-value for the main effect of rate for oxLDL increments, *p* = 0.072, was >0.05 (Supplementary Table S2). There was a significant main effect of time for CD/ApoB-100 concentrations and increments but no other significant main effect or interaction (Supplementary Table S2, Figure 2C and Table 3).

### 3.5. Cardiovascular Hemodynamics

**Figure 3:**
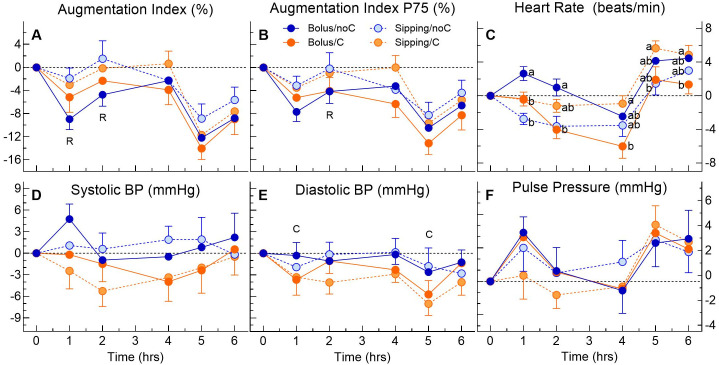
Cardiovascular hemodynamics. Values are means ± SEM for n = 16 subjects after consuming 75 g dextrose within 5 min without (bolus/noC, dark blue) or with 1 g vitamin C (bolus/C, dark orange) or 75 g dextrose sipped evenly over 3.25 h without (sipping/noC, light blue) or with 1 g vitamin C (sipping/C, light orange). Panels: (**A**) increments of AugIdx (%); (**B**) increments of AIp75 (%); (**C**) increments of heart rate (BPM); (**D**) increments of SBP (mmHg); (**E**) increments of DBP (mmHg); (**F**) increments of PP (mmHg). R = significant main effect of rate (*p* < 0.05). C = significant main effect of vitamin C (*p* < 0.05). ^ab^ significant rate × vitC interaction; means not sharing the same letter differ significantly by Tukey’s test, *p* < 0.05.

The main effects of time and rate were significant for AugIdx and AIp75 values and increments (Supplementary Table S2). There was a main effect of rate for AugIdx at 1 and 2 h and for AIp75 at 2 h (Figure 3A,B). For AugIdx and AIp75, nAUC04 and nAUC0-6 were significantly higher after sipping than bolus, and nAUC4b6 was significantly higher after vitamin C than no vitamin C (Table 4).

The main effect of time was significant for heart rate (BPM) values and increments, and there was a significant rate × vitC interaction for BPM increments (Supplementary Table S2). There were significant rate × vitC interactions at every time point (Figure 3C), with BPM after bolus higher than after bolusC and sipping at 1 and 2 h, while, from 4 to 6 h, BPM after sippingC was higher than bolusC. There were also significant rate × vitC interactions for nAUC04 (bolus > bolusC and sipping) and nAUC06 (bolus > bolusC; Table 4).

There was a significant main effect of rate for SBP values but no significant main effects or interactions for SBP increments (Supplementary Table S2). The mean SBP increments did not differ among the treatments at any time point (Figure 3D) or for any AUC (Table 4).

The main effect of time was significant for DBP values and increments, with a significant main effect of vitamin C for DBP increments (Supplementary Table S2). This is reflected in significantly lower DBP increments after vitamin C vs. no vitamin C at 1 h and 5 h (Figure 3E) and a lower DBP nAUC06 after vitamin C versus no vitamin C (Table 4).

There was a significant main effect of time for PP values and increments (Supplementary Table S2), with PP peaking at 1 h and 5 h, but there were no significant differences among the treatments at any point in time (Figure 3F) nor for any AUC (Table 4).

### 3.6. Blood Lipids

There was a significant main effect of time on cholesterol, triglycerides, LDL cholesterol and HDL cholesterol concentrations and increments; however, there were no other significant main effects or interactions (Supplementary Table S2) observed. Thus, the results of statistical analysis of values at individual time points and AUCs are not considered to be conclusive and are not discussed further here. Nevertheless, they are presented in the Supplementary Materials (Table S3, Figure S2).

### 3.7. Determinants of TRAP AUC

For AUC04 and AUC06, insulin was strongly positively associated with TRAP, with a modest negative association between TRAP and glucose and a weak association between TRAP and vitamin C, with the overall models being highly significant (*p* < 0.0001) and explaining about 32% of the variation in TRAP. For AUC4b6, only insulin was weakly negatively associated with TRAP (Table 5).

### 3.8. Determinants of oxLDL AUC

For AUC04, TRAP was moderately negatively associated with oxLDL (*p* = 0.023), but, for AUC06, the negative association between TRAP and oxLDL was strong and explained 20% of the variation in oxLDL. For AUC4b6 glucose was weakly associated with oxLDL (Table 5). The AUCs for vitamin C were not significantly associated with those for oxLDL.

### 3.9. Determinants of CD/ApoB-100 AUC

For AUC04 vitamin C was moderately negatively associated with CD/apoB-100 (*p* = 0.014). For AUC06 the very weak negative association between vitamin C and CD/apoB (*p* = 0.13) became stronger when glucose was added to the model (*p* = 0.044) (Table 5). For AUC4b6, vitamin C was negatively associated with CD/apoB-100 (Table 5). The AUCs for TRAP were not significantly associated with those for CD/apoB-100.

## 4. Discussion

The design of this study was based on a protocol developed by Jenkins et al. [10], showing that sipping a solution containing 50 g glucose slowly over 3 h, as compared to bolus consumption over 5 min, reduced postprandial glucose excursions and enhanced insulin economy and glucose disposal. We used this protocol to test the hypothesis that flattening postprandial glycemic excursions reduces oxidative stress because it isolates the rate of nutrient delivery and eliminates common confounders introduced by food-based interventions, such as vitamins, minerals and other food components, which may have antioxidant activity. The results for the primary outcome supported the hypothesis since the mean serum TRAP increment 2 h after lunch after the sipping/noC treatment was significantly higher than that after the bolus/noC treatment; furthermore, there was a main effect of rate for the incremental area under the TRAP curve after lunch (nAUC4b6 using the value before lunch as the baseline), with the mean for sipping being higher than that after bolus. The increase in nAUC4b6 elicited by the sipping vs. the bolus treatments (mean ± SEM), 104 ± 40 μmol·min/L, was similar to that elicited by the mean of the treatments with 1 g of vitamin C vs. those without vitamin C, 171 ± 37 μmol·min/L (*p* = 0.20).

Enzymatic (e.g., superoxide dismutase and glutathione peroxidase) and non-enzymatic (e.g., dietary vitamin E, β-carotene, vitamin C, thiols, and uric acid) defenses work to ensure that sufficient antioxidant capacity is available during times of stress [30,31,32]. While the enzymatic defenses are active in cells, the non-enzymatic antioxidants are present in the plasma, with uric acid and protein thiol groups being key contributors to overall plasma antioxidant capacity [30,31]. However, an increase in antioxidant capacity in plasma is not always desirable [32], and a decrease in antioxidant capacity in plasma or serum is not necessarily undesirable when this decrease corresponds to a reduced level of ROS [33]. The complexity of interpretation of these assays and metabolic pathways speaks to the importance of conducting multiple assays to measure different antioxidant–oxidant outcomes. For this reason, we included several markers of oxidative stress in the plasma: TRAP, LDL oxidation (oxLDL), LDL peroxidation (CD/ApoB-100) and vitamin C.

The presence of an endogenous antioxidant mechanism is well documented in the literature, whereby an acute increase in oxidative stress results in a systemic adaptation in defense. For example, heavy endurance exercise causes oxidative stress, including ROS generation; a marathon run has been shown to increase serum TRAP due to an acute increase in plasma uric acid and the synergistic effects of the various antioxidants present in human plasma [34]. Meal-induced oxidative stress was found to be accompanied by the activation of endogenous antioxidant mechanisms, primarily in the form of increased plasma uric acid and protein thiols, without altering plasma vitamin and carotenoid concentrations [35,36]. This is consistent with our finding regarding the TRAP AUC over the first 4 h (AUC04). This parameter was not significantly different among the treatments, but the increase in TRAP AUC04 elicited by every treatment was indeed significantly different from time zero. Such initial increases in antioxidant capacity may, at first glance, seem counterintuitive, but they also exemplify the requirement to maintain homeostasis and are conceptually similar to the milieu intérieur coined by Claude Bernard [37]. Regression analysis showed that the strongest and most significant determinant of TRAP AUC04 and AUC06 was the positive relationship with the respective insulin AUC (*p* < 0.001). While correlations do not prove causality, this unexpected result is consistent with recent evidence that, in the absence of diabetes, insulin increases antioxidant defenses in various experimental models [38,39]. The regression model showed that TRAP AUC04 was negatively related to glucose and vitamin C AUCs, suggesting that high glucose reduced TRAP by inducing oxidative stress and that vitamin C, when present at high levels, replaced other types of antioxidant defenses.

The negative correlation between TRAP and oxLDL could reflect a reduction in the oxidation of LDL’s polyunsaturated fatty acids attributed to the aforementioned endogenous antioxidant mechanism, which serves to stabilize unsaturated lipids [31]. Vitamin C supplementation did not affect oxLDL (regardless of administration rate), perhaps providing additional support for an endogenous antioxidant response (e.g., protein thiols or uric acid). Conversely, the CD/apoB-100 AUC was negatively related to the vitamin C AUC. Vitamin C, a water-soluble vitamin and nutrient antioxidant, is a well-known and potent protector of vitamin E. Vitamin E has been recognized for its role in prevention/reduction of LDL/lipid oxidation. In this context it is not clear why oxLDL was associated with TRAP but not vitamin C, while CD/apoB-100 was associated with vitamin C but not TRAP.

TRAP AUC04 tended to be lower after sipping than bolus and lower after vitamin C than no vitamin C; however, these differences were not significant. The main effects of rate (bolus vs. sipping) and vitamin C on TRAP were not evident until after lunch, when TRAP AUC4b6 was significantly higher after sipping than bolus after vitamin C than no vitamin C (Table 3, Figure 2A). The effects of glucose intake rate and vitamin C did not differ significantly (*p* = 0.20), suggesting that sipping glucose reduced oxidative stress to the same extent as 1 g of oral vitamin C and that vitamin C supplementation does not provide additional benefits to a system that already has sufficient protection (antioxidants) against oxidation (sipping). These hypotheses are in agreement with existing data on the effect of vitamin C on postprandial response to OGTT and other acute nutrient loads (e.g., ultra-high-fat meal to induce hyperlipidemia), leading to vitamin C being regarded as protective [30,40,41,42].

Ascorbic acid is generally regarded as a potent antioxidant that improves endothelium–nitric oxide-dependent vasodilation in vascular beds of patients with conditions characterized by endothelial dysfunction: hypertension, DM, hypercholesterolemia, and coronary heart disease [40,41,42]. This is consistent with the significantly greater reduction in AugIdx and AIp75 (measures of arterial stiffness) after lunch (i.e., nAUC4b6) for the vitamin C treatments vs. the no vitamin C treatments (Figure 3A,B and Table 4). However, the fact that AugIdx and AIp nAUC04 and nAUC06 were significantly lower after bolus than sipping (main effect of rate) may reflect the increase in TRAP after all the treatments from times 0 to 4 h. The more rapid the delivery of nutrients to the stomach, the greater the increase in the blood flow needed to support the increased motility required to deliver the nutrients to the small intestine, and the greater the increase in blood flow to the small intestine and other organs involved in the digestion and absorption of the test meal. This may explain the greater increase in pulse rate at 1 h after bolus vs. sipping (the difference being attenuated by bolus/C) and the increase in heart rate after lunch, with the increase from the pre-lunch value being significantly less than after vitamin C vs. no vitamin C. The designs of two previous studies on the acute effects of vitamin C on hemodynamics differ from ours in that the participants took 2 g vitamin C alone and did not eat anything for the 6–8 h duration of the studies [43,44]. This may explain why one of these studies showed that vitamin C significantly reduced AugIdx [43] but the other did not [44].

Administration of vitamin C had no significant effect on SBP increments but had a main effect on DBP increments, reducing DPB 1 h after the start of each eating occasion (glucose consumption at 0 h and lunch at 4 h) and reducing overall nAUC0-6. These effects may reflect an effect of vitamin C on arterial stiffness. Other studies have shown that vitamin C acutely reduced mean arterial pressure [43] or SBP (but not DBP) [44], but these changes were not always associated with a reduction in AugIdx.

This study has potential weaknesses. The number of subjects was sufficient to find a statistically significant effect of the rate of glucose consumption on postprandial TRAP but too small for some secondary endpoints and multiple regression analyses, the results of which should be considered as hypothesis-generating. The single blinding of the study is a potential source of bias. The participants and those administering the treatments could not be blinded to treatment (Sipping vs. Bolus). However, single blinding was maintained by coding the treatments (A, B, C, and D) and identifying them for the co-authors doing the biochemical (K.A.M.-B., D.K., and D.E.B.) and statistical analyses (T.M.S.W.) only by code. The specific endpoint was not specified a priori, and two main statistical analyses were performed (e.g., TRAP concentrations vs. increments). This could lead to potential bias and type 1 error. However, the *p*-value for rate × time interaction for TRAP increments, 0.016, would still be <0.05 if multiplied by 2 (Bonferroni correction).

This study provides additional data on the relationship between slowing carbohydrate absorption and postprandial oxidative/antioxidant bursts and arterial stiffness. The findings are consistent with those from previous studies where low-GI test meals and diets reduced oxidative stress or conserved endogenous antioxidant capacity when compared to high-GI diets [16,17]. However, our results also highlight the potential role of endogenous antioxidants [34,35,36] in regulating postprandial oxidative stress, which is information that is useful for future hypothesis generation and study development. The data provide a rationale to further develop the methodology used to test the effect of slowing carbohydrate delivery on postprandial oxidative response. Outcomes including protein thiols and uric acid will likely provide more insight into the relationship between TRAP, LDL oxidation and acute hyperglycemia. Vitamin E is another biochemical outcome that could provide more information on the relationship between vitamin C and onset of LDL oxidation. Educators and clients/patients should be consulted on their interest, ability and suggestions about how to translate these and future antioxidant GI research findings into practice [45].

## 5. Conclusions

We conclude that the results of this study support the hypothesis that reducing postprandial glycemic excursions reduces oxidative stress. Sipping glucose slowly over 3.5 h, compared to bolus glucose consumption, increased plasma TRAP nAUC after lunch to a similar extent as adding 1 g of vitamin C to the bolus glucose drink.

## Figures and Tables

**Table 1:** Blood sample collection tubes, endpoints and times of collection.

Tube Type (Additive) *	Tube Volume	Analyte	Time (min)								
			0	30	60	120	180	240	270	300	360
Lavender-top (K_2_ EDTA for clot prevention)	10 mL	TRAP	X			X	X	X		X	X
		oxLDL	X			X	X	X		X	X
		CD/ApoB-100	X			X	X	X		X	X
Gold-top (Clot activator gel for serum separation)	6 mL	Glucose	X	X	X	X	X	X	X	X	X
		Insulin	X	X	X	X	X	X	X	X	X
		FFA	X		X	X	X	X		X	X
Green-top (Heparin)	5 mL	Vitamin C	X			X	X	X		X	X

* Vacutainer^®^ Blood Collection Tubes, BD-Canada, Mississauga, ON. X = blood collection. EDTA = ethylenediaminetetraacetic acid; TRAP = total peroxyl radical trapping antioxidant potential (primary endpoint); oxLDL = oxidized low-density lipoprotein cholesterol; CD/ApoB-100 = conjugated dienes per Apolipoprotein B-100; FFA = free fatty acid.

**Table 2:** AUCs and maximum excursions for glucose, insulin, FFA and vitamin C.

Endpoint (Units)	Measure	Individual Treatments				Means for Main Effects			
		Bolus/noC (B)	Bolus/C (BC)	Sipping/noC (S)	Sipping/C (SC)	Bolus (B + BC)	Sipping (S + SC)	No VitC (B + S)	VitC (BC + SC)
Glucose (AUC, mmol·min/L; MAE, mmol/L)	iAUC04	332 ± 43	305 ± 42	256 ± 32	281 ± 27	319 ± 40	269 ± 23	294 ± 28	293 ± 30
	iAUC06	403 ± 43	384 ± 46	308 ± 36	365 ± 36	394 ± 40	337 ± 25	355 ± 27	375 ± 35
	iAUC4b6	171 ± 18	166 ± 18	22 ± 11	36 ± 17	169 ± 12	29 ± 10 *	97 ± 11	101 ± 13
	MAE	5.7 ± 0.5	5.4 ± 0.4	3.9 ± 0.4	4.2 ± 0.4	5.6 ± 0.4	4.0 ± 0.4 *	4.8 ± 0.4	4.8 ± 0.3
Insulin (AUC, pmol·h/L; MAE, pmol/L)	iAUC04	688 ± 109	615 ± 60	452 ± 72	487 ± 74	651 ± 74	470 ± 68	570 ± 73	551 ± 59
	iAUC06	1110 ± 171	1069 ± 108	866 ± 119	942 ± 123	1089 ± 114	904 ± 114	988 ± 126	1005 ± 91
	iAUC4b6	348 ± 41	383 ± 40	135 ± 28	184 ± 37	366 ± 32	160 ± 24 *	242 ± 23	284 ± 31
	MAE	614 ± 91	577 ± 59	337 ± 46	386 ± 79	596 ± 70	361 ± 59 *	475 ± 63	481 ± 63
FFA (AUC, mEQ·min/L; MAE, mEQ/L)	nAUC04	−59 ± 8	−63 ± 9	−59 ± 10	−70 ± 8	−61 ± 7	−64 ± 8	−59 ± 7	−66 ± 8
	nAUC06	−77 ± 12	−79 ± 14	−90 ± 14	−107 ± 13	−78 ± 10	−99 ± 12	−83 ± 11	−93 ± 11
	nAUC4b6	−22 ± 4	−21 ± 5	−1 ± 3	−1 ± 3	−22 ± 4	−1 ± 2 *	−11 ± 2	−11 ± 3
	MAE	0.50 ± 0.04	0.53 ± 0.06	0.38 ± 0.05	0.42 ± 0.04	0.52 ± 0.04	0.40 ± 0.04 *	0.44 ± 0.04	0.48 ± 0.04
Vitamin C (AUC, μmol·min/L; MAE, μmol/L)	nAUC04	−4 ± 2	72 ± 16	4 ± 3	82 ± 12	34 ± 8	43 ± 6	0 ± 2	77 ± 13 *
	nAUC06	−6 ± 4	140 ± 24	4 ± 5	67 ± 12		75 ± 7	−1 ± 3	143 ± 18 *
	nAUC4b6	0 ± 1	−6 ± 4	0 ± 2	−3 ± 2		0 ± 2	0 ± 1	−3 ± 3
	MAE	7 ± 1	42 ± 5	9 ± 2	25 ± 3		25 ± 2	8 ± 1	42 ± 4 *

Values are means ± SEM for n = 16 subjects. iAUC = incremental area under the curve (area below baseline ignored); nAUC = net incremental AUC (negative area below baseline included); AUC04 and AUC06 = AUC calculated from 0 to 4 h and 0 to 6 h, respectively, using the fasting (0 min) value as baseline; AUC4b6 = AUC calculated from 4 to 6 h using the value at 4 h (before lunch) as baseline; MAE = maximum amplitude of excursion (maximum minus minimum concentration from 0 to 6 h). * Significant main effect: *p* < 0.05 (orange); *p* < 0.01 (blue); *p* < 0.001 (green).

**Table 4:** AUCs for cardiovascular hemodynamics.

Endpoint (Units)	Measure	Individual Treatments				Means for Main Effects			
		Bolus/noC (B)	Bolus/C (BC)	Sipping/noC (S)	Sipping/C (SC)	Bolus (B + BC)	Sipping (S + SC)	No VitC (B + S)	VitC (BC + SC)
Augmentation Index: (AugIdx, %·h/L)	iAUC04	−15.9 ± 6.5	−11.8 ± 7.1	−1.8 ± 7.4	−2.5 ± 5.4	−13.8 ± 5.4 *	−2.1 ± 4.6	−8.9 ± 5.9	−7.1 ± 5.1
	iAUC06	−34.8 ± 11.1	−33.0 ± 10.7	−14.7 ± 12.0	−17.8 ± 8.6	−33.9 ± 8.6 *	−16.3 ± 7.5	−24.7 ± 9.6	−25.4 ± 7.7
	iAUC4b6	−13.2 ± 3.7	−12.7 ± 2.9	−8.3 ± 1.8	−16.5 ± 2.0	−12.9 ± 3.0	−12.4 ± 1.6	−10.7 ± 2.1 *	−14.6 ± 2.0
Normalized AugIdx (AIp75, %·h/L)	iAUC04	−16.1 ± 6.9	−16.6 ± 6.7	−6.8 ± 6.6	−4.6 ± 5.4	−16.3 ± 5.4 *	−5.7 ± 4.1	−11.5 ± 5.6	−10.6 ± 4.9
	iAUC06	−32.6 ± 11.7	−38.1 ± 9.9	−19.7 ± 10.7	−17.4 ± 8.6	−35.3 ± 8.6 *	−18.5 ± 6.6	−26.1 ± 9.0	−27.8 ± 7.4
	iAUC4b6	−8.9 ± 3.5	−7.8 ± 2.9	−4.7 ± 1.7	−12.5 ± 2.0	−8.4 ± 3.0	−8.6 ± 1.6	−6.8 ± 2.0 *	−10.1 ± 1.8
Heart Rate—BPM (beats/min)	nAUC04	92 ± 170 ^a^	−700 ± 207 ^b^	−661 ± 178 ^b^	−180 ± 138 ^ab^	−304 ± 133	−421 ± 127	−285 ± 123	−440 ± 127
	nAUC06	407 ± 280 ^a^	−774 ± 350 ^b^	−634 ± 293 ^ab^	265 ± 222 ^ab^	−184 ± 233	−185 ± 216	−113 ± 203	−255 ± 215
	nAUC4b6	605 ± 70	697 ± 97	495 ± 91	569 ± 89	651 ± 55	532 ± 67	550 ± 62	633 ± 74
Systolic Blood Pressure (SBP: mmHg·h)	iAUC04	2.8 ± 7.1	−6.4 ± 7.9	3.8 ± 7.4	−13.8 ± 6.4	−1.8 ± 6.4	−5.0 ± 4.4	3.3 ± 5.4	−10.1 ± 5.8
	iAUC06	4.5 ± 12.5	−10.5 ± 13.5	6.6 ± 12.0	−17.7 ± 10.2	−3.0 ± 11.4	−5.6 ± 6.8	5.5 ± 9.1	−14.1 ± 9.8
	iAUC4b6	2.7 ± 3.3	3.8 ± 2.7	−1.0 ± 2.6	2.8 ± 3.9	3.3 ± 2.9	0.9 ± 2.2	0.8 ± 2.2	3.3 ± 2.9
Diastolic Blood Pressure (DBP: mmHg·h)	iAUC04	−2.1 ± 4.3	−7.6 ± 6.3	−2.1 ± 5.9	−12.3 ± 4.1	−4.9 ± 4.9	−7.2 ± 3.5	−2.1 ± 3.3	−10.0 ± 4.0
	iAUC06	−5.5 ± 6.7	−15.2 ± 9.9	−5.2 ± 10.0	−22.8 ± 6.1	−10.3 ± 7.6	−14.0 ± 5.6	−5.3 ± 5.2 *	−19.0 ± 6.1
	iAUC4b6	−3.0 ± 2.3	−2.9 ± 2.6	−3.4 ± 1.8	−4.7 ± 2.3	−3.0 ± 2.1	−4.0 ± 1.5	−3.2 ± 1.4	−3.8 ± 2.0
Pulse Pressure (PP: mgHg·h)	nAUC04	4.9 ± 5.8	4.5 ± 6.3	5.9 ± 5.7	−1.8 ± 4.1	4.7 ± 4.5	2.0 ± 2.5	5.4 ± 4.4	1.4 ± 4.3
	nAUC06	9.9 ± 9.3	9.9 ± 9.5	11.8 ± 8.7	4.8 ± 6.5	9.9 ± 7.1	8.3 ± 3.5	10.8 ± 7.1	7.4 ± 6.5
	nAUC4b6	6.6 ± 2.8	6.8 ± 2.2	2.4 ± 1.8	7.5 ± 2.2	6.7 ± 2.3	5.0 ± 1.2	4.5 ± 1.7	7.2 ± 1.8

Values are means ± SEM for n = 15 (AugIdx, AIp75, BPM) or n = 16 (SBP, DBP, PP) subjects. iAUC = incremental area under the curve (area below baseline ignored); nAUC = net incremental AUC (negative area below baseline included); AUC04 and AUC06 = AUC calculated from 0 to 4 h and 0 to 6 h, respectively, using the fasting (0 min) value as baseline; AUC4b6 = AUC calculated from 4 to 6 h using the value at 4 h (before lunch) as baseline. * Significant effects: *p* < 0.05 (orange); *p* < 0.01 (blue). ^ab^ Significant rate × VitC interaction: means with different letter superscripts differ significantly by Tukey’s test (*p* < 0.05).

**Table 5:** Determinants of TRAP, oxLDL and CD/ApoB-100.

Dependent Variable	Measure * (n of OE)	Scalar (β) *p*-Value	Independent Variables *						Intercept	Model r (*p*)
			Glucose	Insulin		Vitamin C		oxLDL		
TRAP	AUC04	β	−0.60 ± 0.28	0.049 ± 0.011		−1.22 ± 0.64		-	2.21 ± 1.25	0.569 (<0.0001)
	(n = 3)	*p*	0.018	<0.0001		0.045		-	0.08	
	AUC4b6	β	-	−0.022 ± 0.01		-		-	0.69 ± 0.54	0.271 (0.037)
	(n = 4)	*p*	-	0.037		-		-	0.19	
	AUC06	β	−0.69 ± 0.31	0.039 ± 0.010		-		−0.64 ± 0.30	1.58 ± 1.84	0.572 (<0.0001)
	(n = 5)	*p*	0.032	0.0003		-		0.041	0.41	
**Dependent** **Variable**	**Measure** **(n of OE)**	**Scalar (β)** ***p*** **-Value**	Independent Variables *						**Intercept**	**Model r** **(*p*)**
			**TRAP**		**Vitamin C**		**Glucose**			
oxLDL	AUC04	β	−0.070 ± 0.030		-		-		0.23 ± 0.19	0.299 (0.023)
	(n = 6)	*p*	0.023		-		-		0.23	
	AUC4b6	β	-		-		−0.10 ± 0.052		0.18 ± 0.11	0.250 (0.054)
	(n = 4)	*p*	-		-		0.054		0.13	
	AUC06	β	−0.13 ± 0.033		-		-		0.46 ± 0.28	0.452 (0.0003)
	(n = 5)	*p*	0.0003		-		-		0.10	
CD/ApoB-100	AUC04	β	-		−0.11 ± 0.042		-		−0.02 ± 0.05	0.312 (0.014)
	(n = 2)	*p*	-		0.014		-		0.72	
	AUC4b6	β	-		−0.21 ± 0.081		-		0.024 ± 0.016	0.313 (0.014)
	(n = 3)	*p*	-		0.014		-		0.13	
	AUC06	β	-		−0.084 ± 0.041		−0.028 ± 0.025		0.13 ± 0.16	0.302 (0.093)
	(n = 4)	*p*	-		0.044		0.22		0.41	

n of OE = number of outliers excluded from a total of n = 64; TRAP = total peroxyl radical trapping antioxidant potential (µmol/L); oxLDL = oxidized low-density lipoprotein cholesterol (units/L); CD/apoB-100 = conjugated dienes/apolipoprotein B100 (μmol/μmol); AUC = incremental area under the curve (negative area ignored for glucose and insulin; negative area included for other variables); AUC04 and AUC06 are the AUCs calculated from 0 to 4 h and 0 to 6 h, respectively, using the fasting (0 min) value as baseline; AUC4b6 is the AUC calculated from 4 to 6 h using the value at 4 h (before lunch) as baseline. Gray shading indicates the only significant variable. Colors indicate the order in which variables were added to the model: green (first); blue (second); orange (third). * Values for the independent variables are the AUCs calculated in the same way as the respective dependent variable.

## Data Availability

The original contributions presented in this study are included in the article/Supplementary Materials. Further inquiries can be directed to the corresponding author.

## Supplementary Materials

The following supporting information can be downloaded at: https://www.mdpi.com/article/10.3390/antiox15040512/s1, Results: blood lipids; Table S1: Fasting concentrations of the study endpoints prior to the consumption of each treatment; Table S2: Results of analyses of variance of absolute values and increments; Table S3: AUCs and maximum excursions for blood lipids; Figure S1: Study diagram; Figure S2: Serum lipid increments: individual results for the 20 endpoints measured (circulating metabolites and cardiovascular hemodynamics).

## Abbreviations

The following abbreviations are used in this manuscript:

ANOVA	Repeated-measures analysis of variance
ApoB-100	Apolipoprotein B-100
AUC	Total area under the curve
AUC04	AUC from 0 to 4 h using the value at time 0 h as the baseline
AUC06	AUC from 0 to 6 h using the value at time 0 h as the baseline
AUC46	AUC from 4 to 6 h using the value at time 0 h as the baseline
AUC4b6	AUC from 4 to 6 h using the value at 4 h as the baseline
AIp75	Augmentation index normalized to a pulse rate of 75 beats per minute
AugIdx	Augmentation index
BP	Blood pressure
Bolus	Dex consumed within 5 min
Bolus/C	Bolus with 1 g vitamin C added
Bolus/noC	Bolus without vitamin C
BPM	Beats per minute (heart rate)
CD	Conjugated dienes
CD/ApoB-100	CD/ApoB-100 concentration ratio
CRP	C-reactive protein
DPB	Diastolic blood pressure
Dex	75 g dextrose dissolved in 250 mL tap water
FFA	Free fatty acid
GI	Glycemic index
iAUC	Incremental area under the curve ignoring area below the baseline
LDL	Low-density lipoprotein cholesterol
MAE	Maximum amplitude of the excursion
netAUC	Incremental area under the curve subtracting area below the baseline
oxLDL	Oxidized LDL
PP	Pulse pressure
SBP	Systolic blood pressure
Sipping	Dex consumed evenly over 3.25 h
Sipping/C	Sipping with 1 g vitamin C added
Sipping/noC	Sipping without vitamin C
TRAP	Total peroxyl radical trapping antioxidant potential
VitC	Vitamin C
