# Supplementary material for: Effect of the Rate of Glucose Consumption on the Total Peroxyl Radical Trapping Antioxidant Potential (TRAP) of Plasma in Overweight Men and Women: A Randomized Trial
Source: Antioxidants (Basel). 2026 Apr 21;15(4):512. doi: 10.3390/antiox15040512 (PMC13113598; doi:10.3390/antiox15040512)
Supplement: Supplementary file 1 [file antioxidants-15-00512-s001.zip › antioxidants-4211471-supplementary.pdf]

Supplementary Material:

Contents

Table S1: Fasting concentrations of the study endpoints prior to the consumption of each treatment. 2
Table S2. Results of analyses of variance of absolute values and increments 3
Results: Blood Lipids 4
Table S3: AUCs and maximum excursions for blood lipids 5
Figure S1: Study diagram 6
Figure S2: Serum lipid increments. 7
- Raw Data: Glucose (mmol/L) 8
- Raw Data: Insulin (μU/mL) 9
- Raw Data: Free fatty-acids (mEq/L) 10
- Raw Data: Vitamin C (μmol/L) 11
- Raw Data: TRAP (μmol/L) 12
- Raw Data: Oxidized LDL (U/L) 13
- Raw Data: CD/ApoB-100 (μmol) 14
- Raw Data: Augmentation Index (%) 15
- Raw Data: Augmentation Index normalized to a pulse pressure of 75mmHg (%) 16
- Raw Data: Heart Rate (beats/min) 17
- Raw Data: Systolic Blood Pressure (mmHg) 18
- Raw Data: Diastolic Blood Pressure (mmHg) 19
- Raw Data: Pulse Pressure (mmHg) 20
- Raw Data: Cholesterol (mmol/L) 21
- Raw Data: Triglycerides (mmol/L) 22
- Raw Data: HDL-cholesterol (mmol/L) 23
- Raw Data: LDL-cholesterol (mmol/L) 24
- Raw Data: C-reactive protein (mg/L) 25
- Raw Data: Conjugated Dienes (μmol/L) 26
- Raw Data: ApoB-100 (μmol/L) 27

Supplementary Material:

Table S1: Fasting concentrations of the study endpoints prior to the consumption of each treatment.

Endpoint	Treatment				P*
	Bolus	Bolus+C	Sipping	Sipping+C	
Total peroxyl radical trapping antioxidant potential (TRAP; U/L)	1123±67	1110±66	1066±51	1050±47	0.98
Oxidized LDL (U/L)	75±10	75±11	65±6	70±9	0.78
Conjugated dienes/apolipoprotein B100 (CD/apoB; µmol/µmol)	17±1	16±2	16±1	19±2	0.39
Plasma glucose (mmol/L)	5.04±0.11	4.89±0.11	4.98±0.16	4.95±0.12	0.71
Serum Insulin (pmol/L)	76±30	56±20	43±6	43±9	0.64
Free Fatty Acids (mEQ/L)	0.47±0.04	0.51±0.05	0.46±0.05	0.51±0.04	0.96
C-reactive protein (mg/L)	3.17±0.92	2.90±0.88	2.33±0.80	2.60±0.99	0.83
Vitamin-C (µmol/L)	18±2	21±3	16±2	19±2	0.98
Total Cholesterol[1] (mmol/L)	5.15±0.20	5.34±0.24	5.25±0.24	5.23±0.25	0.75
Triglyderides[1] (mmol/L)	1.38±0.20	1.51±0.21	1.44±0.22	1.29±0.17	0.62
HDL-cholesterol[1] (mmol/L)	1.32±0.07	1.31±0.07	1.30±0.06	1.28±0.06	0.95
LDL-cholesterol[1] (mmol/L)	3.21±0.21	3.34±0.23	3.30±0.21	3.37±0.23	0.91
Augmentation index[2] (AugIdx; %/L)	81±4	78±5	81±5	82±4	0.74
AI at pulse rate 75 beats/min[2] (AIp75; %/L)	78±4	77±5	79±5	79±4	0.89
Heart rate[2] (BPM; beats/min)	69±2	73±2	70±2	68±2	0.30
Diastolic blood pressure (DBP; mmHg)	76±3	77±3	75±3	76±3	0.94
Systolic blood pressure (SBP; mmHg)	123±3	126±3	121±3	124±4	0.96
Pulse pressure (PP; mmHg)	48±2	48±2	46±2	48±2	63

Values are means±SEM.
* p-value for the rate×vitamin-C interaction. There were no significant main effects of rate or vitamin-C for any endpoint (not shown).
[1] n=14
[2] n=15

Supplementary Material:

Table S2. Results of analyses of variance of absolute values and increments

Endpoint	Concentrations / Absolute Values							Increments						
	Time	Rate	R×T	VitC	V×T	R×V	T×R×V	Time	Rate	R×T	VitC	V×T	R×V	T×R×V
TRAP	dark green				light green			dark green		light green		light green		
oxLDL									light red					
CD/apoB	light green							light green						
Glucose	dark green		dark green					dark green		dark green				
Insulin	dark green	dark green	dark green					dark green	light red	light green				
FFA	dark green	dark green	dark green					dark green	light red	dark green				
CRP														
VitC	dark green			dark green	dark green			dark green			dark green	dark green		
TChol[1]	dark green							dark green						
TG[1]	dark green							dark green						
HDL[1]	dark green							dark green						
LDL[1]	dark green							dark green						
AugIdx[2]	dark green	light green						dark green	light green					
AIp75[2]	dark green	light green						dark green	light green					
BPM[2]	dark green	light red						dark green					light green	
DBP	light green	light red						dark green			light green			
SBP		light green												
PP	dark green							dark green						

TRAP = total peroxyl radical trapping antioxidant potential
oxLDL = oxidized low-density lipoprotein cholesterol
CD/apoB = conjugated dienes/apolipoprotein B100
FFA = free fatty acids
CRP = c-reactive protein
VitC = vitamin C
TChol = total cholesterol
TG = triglycerides
HDL = high-density lipoprotein cholesterol
LDL = low-density lipoprotein cholesterol
AugIdx = augmentation index
AIp75 = augmentation index normalized to heart rate of 75 beats/min
BPM = heart rate in beats per minute
DBP = diastolic blood pressure
SBP = systolic blood pressure
PP = pulse pressure

Color-code: white = $p>0.1$; light red = $0.1>p>0.05$; light green = $p<0.05$; dark green = $p<0.001$,

[1] n=14 subjects
[2] n=15 subjects

Results: Blood Lipids

There was a main effect of time for cholesterol, triglycerides, LDL-cholesterol and HDL-cholesterol concentrations and increments, however, there were no other significant main effects or interactions (Supplementary Table S2). Thus, the results of statistical analysis of values at individual time points and AUCs are not considered to be conclusive. Nevertheless, they are presented here.

Serum total cholesterol (Chol) decreased from baseline during the 6-hour study period with nAUC04 and nAUC06 being significantly less than zero for each of the 4 treatments (Table S3). The Chol increment after Bolus+C was significantly less than that after Bolus at 4hr and significantly less than that after Sipping+C at 6hr (Figure S1A). There rate×vitamin-C interactions for nAUC04 (p=0.056) and nAUC06 (p=0.053) just missed the criterion for statistical significance. However, the rate×vitamin-C interaction for MAE (maximum amplitude of the excursion) was significant (p=0.009) with Bolus+C > Bolus (Table S3).

Serum triglycerides (TG) tended to fall over the first 4h, then increased significantly after lunch (Table S3 and Figure S1B). There was a significant main effect of rate on the post lunch increase (nAUC4B6) and MAE with both being significantly greater after Sipping than Bolus (Table S3).

Serum LDL-cholesterol tended to fall during the day, and particularly after lunch although there was no significant difference among treatments (Figure S1C and Table S3). However, there was a significant rate×vitamin-C interaction (p=0.037) for MAE with that after Bolus+C being greater than that after Bolus (Table S3).

For serum HDL-cholesterol there were a significant rate×vitamin-C interactions for the increment at 2hr (Sipping < Sipping+C, p=0.044, Figure S1D), nAUC04 (Sipping < Sipping+C , p=0.044, Table S3) and MAE (Bolus+C > Sipping+C, p=0.036, Table S3). There was a significant main effect or rate for HDL-cholesterol nAUC06 with Sipping > Bolus (p=0.040, Table S3).

Supplementary Material:

Table S3: AUCs and maximum excursions for blood lipids.

		Individual Treatments				Means for Main Effects			
		Bol-C (B)	Bol+C (BC)	Sip-C (S)	Sip+C (SC)	Bolus (B+BC)	Sipping (S+SC)	No VitC (B+S)	VitC (BC+SC)
Chol (mmol/L)	nAUC04[1]	-0.27±0.09	-0.83±0.28	-0.60±0.13	-0.35±0.12	-0.55±0.16	-0.47±0.09	-0.43±0.08	-0.59±0.16
	nAUC06[1]	-0.47±0.13	-1.47±0.48	-0.91±0.20	-0.58±0.19	-0.97±0.28	-0.74±0.14	-0.69±0.12	-1.02±0.28
	nAUC4b6[1]	-0.17±0.05	-0.04±0.09	0.03±0.10	0.00±0.05	-0.10±0.05	0.01±0.05	-0.07±0.05	-0.02±0.06
	MAE	0.27±0.03[b]	0.53±0.10[a]	0.38±0.05[ab]	0.30±0.03[ab]	0.40±0.06	0.34±0.03	0.32±0.03	0.42±0.05
TG (mmol/L)	nAUC04[2]	-0.35±0.25	-0.57±0.29	-0.17±0.15	-0.45±0.14	-0.46±0.26	-0.31±0.10	-0.26±0.18*	-0.51±0.18
	nAUC06[2]	-0.32±0.49	-0.67±0.53	0.01±0.35	-0.38±0.26	-0.50±0.50	-0.19±0.25	-0.16±0.41*	-0.53±0.33
	nAUC4b6[2]	0.24±0.04	0.35±0.07	0.52±0.15	0.45±0.05	0.30±0.05*	0.48±0.07	0.38±0.08	0.40±0.05
	MAE[2]	0.59±0.10	0.65±0.11	0.91±0.13	0.77±0.07	0.62±0.10*	0.84±0.08	0.75±0.10	0.71±0.08
HDL (mmol/L)	nAUC04	0.00±0.04[ab]	-0.05±0.05[ab]	-0.10±0.02[b]	0.04±0.03[a]	-0.02±0.04	-0.03±0.02	-0.05±0.03	0.00±0.04
	nAUC06[1]	-0.02±0.09	-0.13±0.09	-0.15±0.04	0.05±0.05	-0.07±0.08	-0.05±0.04	-0.08±0.06	-0.04±0.06
	nAUC4b6[2]	-0.07±0.01	-0.06±0.03	-0.02±0.02	-0.03±0.02	-0.07±0.01*	-0.03±0.01	-0.05±0.01	-0.05±0.02
	MAE	0.10±0.01[ab]	0.13±0.02[a]	0.09±0.01[ab]	0.07±0.01[b]	0.11±0.01	0.08±0.01	0.09±0.01	0.10±0.01
LDL (mmol/L)	nAUC04[1]	-0.12±0.11	-0.52±0.24	-0.42±0.09	-0.18±0.10	-0.32±0.15	-0.30±0.09	-0.27±0.08	-0.35±0.16
	nAUC06[1]	-0.31±0.19	-1.03±0.42	-0.76±0.20	-0.45±0.16	-0.67±0.27	-0.61±0.16	-0.54±0.17	-0.74±0.27
	nAUC4b6[1]	-0.20±0.05	-0.14±0.06	-0.19±0.09	-0.17±0.04	-0.17±0.03	-0.18±0.05	-0.20±0.06	-0.15±0.04
	MAE	0.30±0.04[b]	0.50±0.08[a]	0.42±0.07[ab]	0.33±0.04[ab]	0.40±0.05	0.38±0.05	0.36±0.04	0.42±0.05

Values are means±SEM for n=14 subjects. Chol = total cholesterol; TG = triglycerides; HDL = high-density lipoprotein cholesterol; LD = low-density lipoprotein cholesterol; nAUC = net incremental area under the curve (negative area below baseline included); nAUC04 and nAUC06 are the nAUC calculated from 0-4h and 0-6h, respectively, using the fasting (0min) value as the baseline; nAUC4b6 is the AUC calculated from 4-6h using the value at 4h (before lunch) as the baseline.

* Significant main effect: $p<0.05$ (orange shading)

[ab] Significant rate×VitC interaction: means with different letter superscripts differ significantly by Tukey's test ($p<0.05$) (blue shading).

[1] No significant main effects and no significant rate×vitC interaction.

[2] No significant rate×vitC interaction.

Supplementary Material:

Figure S1: Study diagram

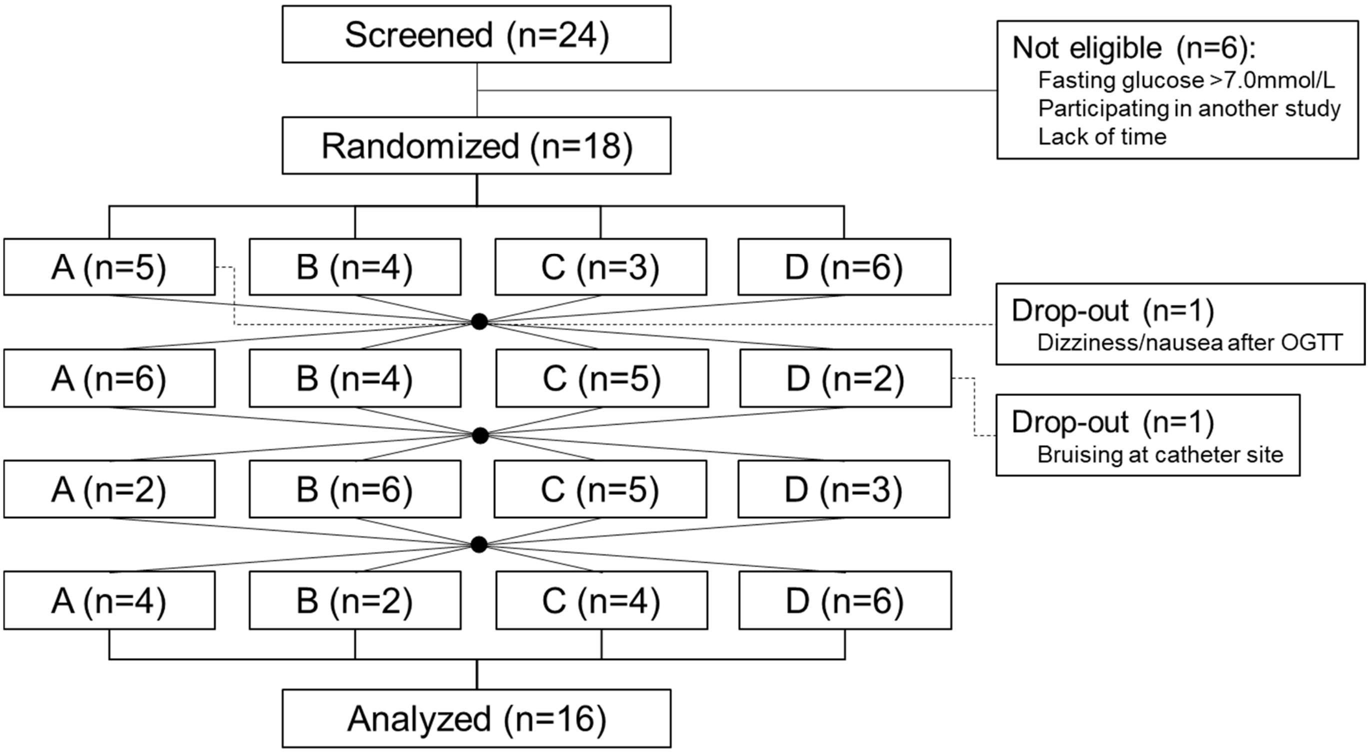

A = Bolus without vitamin C

B = Bolus with vitamin C

C = Sipping without vitamin C

D = Sipping with vitamin C

Supplementary Material:

Figure S2: Serum lipid increments.

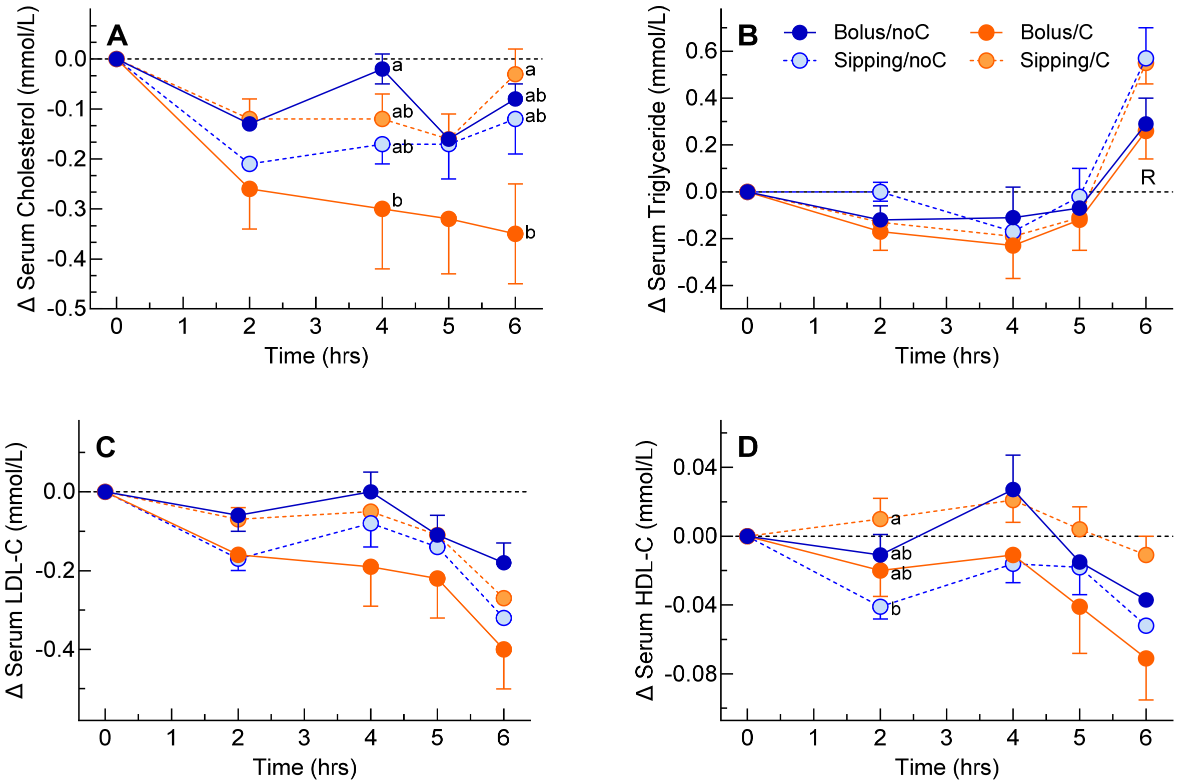

Values are means±SEM for n=14 subjects after consuming 75g dextrose within 5min (Bolus, dark blue circles) or 75g dextrose evenly over 3.25h (Sipping, light blue circles), or Bolus plus 1g vitamin-C (Bolus+C, dark orange circles) or Sipping plus 1g vitamin-C (Sipping+C, light orange circles). Panels: A, serum total cholesterol increments (mmol/L); B, serum triglyceride increments (mmol/L): C, serum LDL-cholesterol increments (mmol/L); D, serum HDL-cholesterol increments (mmol/L).

R = significant main effect of rate ($p<0.05$).

[ab] significant rate×vitamin-C interaction; means not sharing the same letter differ significantly by Tukey's test, $p<0.05$.

Supplementary Material:

Raw Data: Glucose (mmol/L)

ID	Treatment	0min	30min	60min	120min	180min	240min	270min	300min	360min
102	Bolus	5.72	9.27	11.10	6.78	3.20	3.59	4.42	6.59	6.99
209	Bolus	5.07	8.60	8.09	6.61	5.50	4.55	4.65	6.12	6.30
258	Bolus	5.78	10.20	11.80	9.57	5.32	3.32	3.58	5.91	3.35
299	Bolus	4.84	7.97	8.14	5.67	4.14	4.01	4.31	6.62	6.25
333	Bolus	4.78	7.75	6.40	6.27	3.82	3.84	4.52	4.82	6.14
341	Bolus	5.44	10.80	10.90	5.83	3.86	4.33	5.50	5.86	5.18
354	Bolus	4.63	6.73	4.74	6.37	4.71	4.05	5.30	4.94	5.16
390	Bolus	4.57	8.37	6.65	5.01	2.74	4.20	4.73	6.83	5.55
393	Bolus	4.87	6.26	4.66	4.13	4.41	4.67	4.84	5.61	3.69
396	Bolus	4.96	8.92	10.60	4.42	3.98	4.25	4.24	5.37	4.83
399	Bolus	5.34	10.40	8.44	6.28	4.49	4.07	5.69	6.29	6.04
436	Bolus	5.42	10.60	10.50	6.65	3.43	4.02	4.45	6.60	5.72
458	Bolus	4.91	7.93	5.93	6.85	3.92	4.22	4.97	6.95	5.80
519	Bolus	5.05	9.07	10.80	7.41	2.99	4.00	4.72	7.93	6.07
528	Bolus	4.14	5.52	6.26	4.58	5.12	3.00	3.70	6.49	5.60
529	Bolus	5.14	8.45	12.30	9.81	6.84	2.79	4.37	5.92	6.09
102	Bolus + Vit C	5.43	9.73	10.30	6.77	3.22	4.09	4.15	5.12	5.55
209	Bolus + Vit C	4.81	6.20	7.35	5.22	4.08	3.73	4.00	5.20	5.12
258	Bolus + Vit C	5.97	10.50	10.30	9.27	6.03	4.71	4.67	7.45	6.78
299	Bolus + Vit C	5.10	8.15	6.09	5.76	2.80	3.96	4.27	6.23	6.11
333	Bolus + Vit C	5.02	8.15	7.55	6.44	4.81	3.63	4.43	6.74	6.18
341	Bolus + Vit C	4.51	8.24	7.14	2.88	3.61	4.49	5.23	5.29	5.68
354	Bolus + Vit C	4.57	7.57	6.02	5.70	3.17	4.74	3.99	7.30	5.30
390	Bolus + Vit C	4.71	6.12	6.82	5.32	3.09	4.37	4.76	5.54	5.06
393	Bolus + Vit C	4.20	8.33	7.20	4.71	2.92	3.57	4.74	6.84	5.71
396	Bolus + Vit C	4.71	8.30	8.90	4.07	3.99	3.76	4.92	4.24	5.35
399	Bolus + Vit C	4.81	8.60	8.13	5.57	4.40	3.14	4.90	5.08	5.88
436	Bolus + Vit C	5.19	8.24	10.80	7.06	2.81	3.31	3.72	7.52	6.44
458	Bolus + Vit C	5.14	8.22	9.56	5.98	4.65	3.97	4.61	6.02	6.23
519	Bolus + Vit C	4.98	9.88	9.70	7.76	2.60	4.13	5.12	7.61	6.98
528	Bolus + Vit C	4.40	5.64	4.55	4.37	3.34	3.73	4.22	4.77	5.06
529	Bolus + Vit C	4.69	8.08	9.34	10.60	6.03	3.92	2.97	5.77	5.58
102	Sipping	5.77	10.20	10.20	7.39	4.62	4.21	3.90	4.51	5.63
209	Sipping	4.88	5.34	6.00	6.09	4.56	5.03	5.28	3.44	3.94
258	Sipping	6.77	6.64	9.26	7.20	5.38	8.72	6.22	6.48	5.25
299	Sipping	4.81	5.15	7.00	7.20	5.36	4.92	3.37	5.70	4.54
333	Sipping	4.76	7.08	7.90	7.28	7.82	4.40	4.70	4.99	4.67
341	Sipping	4.99	8.70	10.20	4.83	3.71	6.51	4.10	4.41	4.84
354	Sipping	4.19	6.38	8.84	7.62	4.29	6.03	3.32	5.77	5.10
390	Sipping	4.55	5.61	6.11	4.21	4.53	6.11	3.80	4.58	5.08
393	Sipping	4.38	5.16	5.27	4.32	4.59	4.69	4.05	5.70	4.62
396	Sipping	4.67	5.28	7.52	7.17	4.66	7.08	4.46	4.08	5.01
399	Sipping	5.12	7.89	7.55	5.81	4.28	3.98	5.29	5.77	5.71
436	Sipping	5.32	6.51	6.34	5.54	5.73	6.63	5.98	3.80	5.15
458	Sipping	4.76	7.25	7.48	7.87	6.19	5.61	4.32	6.10	5.90
519	Sipping	5.08	7.38	9.98	6.06	5.42	6.88	4.39	6.23	4.62
528	Sipping	4.30	5.61	5.44	4.79	5.16	4.43	3.74	4.04	4.61
529	Sipping	5.32	6.31	8.04	7.60	6.30	6.96	3.58	5.26	5.21
102	Sipping + Vit C	5.48	6.53	10.40	7.97	7.07	4.73	4.20	5.92	6.21
209	Sipping + Vit C	4.91	7.96	7.35	6.08	3.64	4.89	4.03	4.73	3.72
258	Sipping + Vit C	5.32	6.61	7.32	8.84	5.94	7.56	7.79	6.52	5.64
299	Sipping + Vit C	5.65	8.04	8.89	6.02	4.01	3.35	4.16	6.33	6.18
333	Sipping + Vit C	5.03	7.61	9.52	5.61	4.70	7.00	4.73	5.55	5.59
341	Sipping + Vit C	5.48	10.50	9.87	5.62	3.45	4.83	4.56	4.92	5.65
354	Sipping + Vit C	4.66	5.91	6.00	5.71	5.43	5.73	3.24	3.97	4.90
390	Sipping + Vit C	4.18	4.67	5.80	3.89	3.69	4.87	4.34	4.88	4.86
393	Sipping + Vit C	4.23	5.66	5.37	3.98	4.79	4.68	5.85	3.81	3.75
396	Sipping + Vit C	4.75	6.82	9.53	5.73	7.68	4.53	2.82	5.78	3.95
399	Sipping + Vit C	5.05	7.08	6.59	5.61	6.54	6.82	5.76	5.79	6.23
436	Sipping + Vit C	5.34	5.19	9.03	7.28	7.91	7.04	6.05	6.66	5.14
458	Sipping + Vit C	4.94	7.11	7.29	6.03	5.89	6.52	6.16	4.82	5.87
519	Sipping + Vit C	5.04	7.56	8.18	5.34	6.45	3.54	4.21	5.51	4.65
528	Sipping + Vit C	4.00	5.82	6.39	6.48	5.50	5.27	3.28	4.38	5.43
529	Sipping + Vit C	5.15	6.33	9.24	8.36	6.85	5.79	3.43	5.70	5.82

Supplementary Material:

Raw Data: Insulin (µU/mL)

ID	Treatment	0min	30min	60min	120min	180min	240min	270min	300min	360min
102	Bolus	11.43	91.07	124.48	53.09	3.00	3.00	14.24	102.96	41.04
209	Bolus	6.85	79.96	36.09	101.72	28.71	10.59	14.27	54.39	33.46
258	Bolus	15.97	67.60	128.50	131.76	58.88	16.26	43.88	72.23	23.76
299	Bolus	5.47	56.68	132.56	14.70	7.32	3.00	5.82	46.72	39.63
333	Bolus	3.48	49.74	62.69	24.95	3.79	3.00	4.50	32.54	29.45
341	Bolus	14.68	127.65	308.12	99.89	21.72	8.97	84.27	128.24	29.15
354	Bolus	6.46	62.05	40.62	40.62	14.52	5.85	49.81	47.13	19.89
390	Bolus	4.65	77.52	54.18	29.15	3.00	3.00	3.00	42.29	25.54
393	Bolus	9.08	46.39	35.34	11.26	3.00	4.46	3.57	28.93	35.28
396	Bolus	87.41	14.45	141.46	94.72	25.47	15.54	27.83	67.02	42.73
399	Bolus	4.15	52.36	73.73	20.93	5.65	3.66	23.42	43.95	21.31
436	Bolus	7.20	64.89	115.55	68.82	8.34	4.99	9.54	46.70	25.15
458	Bolus	6.98	38.75	41.07	28.48	3.00	3.00	3.00	34.19	11.47
519	Bolus	6.93	33.39	73.79	47.80	6.39	3.00	3.00	48.12	26.63
528	Bolus	4.64	112.59	57.15	41.95	33.71	3.00	3.00	68.28	39.67
529	Bolus	8.02	46.86	98.70	125.51	88.65	16.31	49.20	70.38	46.65
102	Bolus + Vit C	11.55	80.91	98.87	40.94	12.96	7.19	7.63	62.89	30.53
209	Bolus + Vit C	3.00	22.37	37.46	29.79	6.68	3.00	3.00	29.05	13.73
258	Bolus + Vit C	57.65	92.11	95.82	114.44	37.17	10.99	31.27	75.85	67.19
299	Bolus + Vit C	8.30	105.00	53.11	37.92	5.27	3.60	13.36	34.80	40.18
333	Bolus + Vit C	5.34	61.68	62.34	46.53	6.06	3.00	7.19	56.29	30.39
341	Bolus + Vit C	3.00	105.39	193.32	25.25	3.97	3.00	30.07	68.92	60.60
354	Bolus + Vit C	8.82	110.77	102.42	57.13	3.20	10.99	9.46	136.83	45.31
390	Bolus + Vit C	3.47	33.82	53.22	19.63	3.00	3.00	10.25	36.80	23.90
393	Bolus + Vit C	3.00	50.35	82.68	28.99	3.00	3.00	17.73	61.97	19.22
396	Bolus + Vit C	10.13	81.73	121.98	36.87	21.89	13.88	35.43	67.03	71.78
399	Bolus + Vit C	3.00	59.86	77.99	26.59	8.48	3.19	34.47	30.54	20.93
436	Bolus + Vit C	7.76	46.93	106.78	47.00	8.44	5.77	4.51	89.61	52.24
458	Bolus + Vit C	7.24	42.44	51.47	29.70	7.29	3.00	10.84	36.19	27.39
519	Bolus + Vit C	7.69	97.24	99.39	53.89	8.86	3.00	17.55	103.18	56.44
528	Bolus + Vit C	3.19	143.33	46.18	22.79	10.74	3.00	4.55	27.36	30.29
529	Bolus + Vit C	6.54	36.61	80.04	123.46	58.79	13.87	16.60	69.17	45.77
102	Sipping	11.90	57.71	99.51	53.13	18.00	10.63	13.54	50.96	47.48
209	Sipping	4.66	6.23	13.91	11.26	9.06	6.15	16.49	12.59	7.43
258	Sipping	17.82	21.27	45.21	46.83	16.31	44.21	100.65	84.80	24.13
299	Sipping	5.57	12.41	51.19	35.94	14.22	8.14	3.00	43.74	11.30
333	Sipping	6.99	30.14	26.95	39.79	42.99	5.74	24.08	42.42	23.44
341	Sipping	6.59	78.56	141.66	76.61	13.74	59.47	48.82	63.01	18.36
354	Sipping	8.86	42.46	76.39	85.65	18.75	38.09	11.21	78.49	48.45
390	Sipping	5.15	17.14	26.10	4.15	3.00	21.47	3.00	12.19	12.95
393	Sipping	3.00	28.21	23.23	8.83	3.00	9.75	3.46	39.40	19.10
396	Sipping	10.47	24.93	63.13	77.45	29.42	76.46	39.23	40.75	31.62
399	Sipping	3.00	56.37	70.33	23.50	10.96	3.00	30.21	37.08	16.85
436	Sipping	6.33	14.27	11.39	13.19	10.03	18.72	56.67	19.08	26.26
458	Sipping	3.83	24.49	17.66	57.04	10.18	6.42	3.00	19.22	24.88
519	Sipping	8.56	34.27	54.47	28.45	15.49	23.58	3.70	48.25	29.83
528	Sipping	3.00	20.38	20.44	13.76	18.95	10.49	11.86	16.02	23.01
529	Sipping	9.89	25.28	42.01	48.45	40.43	51.53	20.24	56.24	31.24
102	Sipping + Vit C	9.46	23.89	54.19	48.82	55.77	10.61	12.96	73.73	44.00
209	Sipping + Vit C	3.00	34.21	26.59	23.06	13.23	5.65	3.00	37.17	12.00
258	Sipping + Vit C	24.81	24.94	39.48	65.41	63.01	66.55	129.89	115.64	64.99
299	Sipping + Vit C	11.01	46.33	105.87	45.88	15.24	9.31	8.71	33.59	58.86
333	Sipping + Vit C	6.67	34.93	71.21	19.15	6.14	39.95	11.76	45.14	26.59
341	Sipping + Vit C	12.09	107.14	241.00	69.04	17.56	22.26	19.08	69.27	44.84
354	Sipping + Vit C	6.80	19.59	16.54	14.45	12.10	52.42	17.23	52.29	18.23
390	Sipping + Vit C	3.00	8.39	26.81	11.88	3.00	4.79	10.36	20.03	12.03
393	Sipping + Vit C	3.00	18.14	16.74	5.79	9.71	3.00	23.98	19.30	9.38
396	Sipping + Vit C	10.74	49.41	89.61	64.19	58.20	15.16	10.82	120.94	27.52
399	Sipping + Vit C	3.00	22.06	32.98	11.71	25.94	15.17	24.55	35.28	19.33
436	Sipping + Vit C	4.54	4.32	34.02	37.73	28.35	23.98	24.52	57.49	18.96
458	Sipping + Vit C	4.45	30.34	27.21	26.68	12.96	14.34	19.58	13.69	29.55
519	Sipping + Vit C	3.48	24.72	23.26	10.85	15.75	3.00	4.01	44.54	12.36
528	Sipping + Vit C	3.00	34.23	35.58	31.37	30.43	24.60	14.14	24.22	34.09
529	Sipping + Vit C	6.38	19.44	33.73	54.13	46.38	23.85	13.20	47.07	29.41

Supplementary Material:

Raw Data: Free fatty-acids (mEq/L)

ID	Treatment	0min	30min	60min	120min	180min	240min	270min	300min	360min
102	Bolus	0.573		0.161	0.084	0.234	0.760		0.565	0.172
209	Bolus	0.541		0.057	0.268	0.407	0.830		0.542	0.379
258	Bolus	0.560		0.341	0.136	0.157	0.444		0.552	0.432
299	Bolus	0.502		0.066	0.057	0.144	0.684		0.595	0.087
333	Bolus	0.712		0.104	0.055	0.116	0.709		0.240	0.093
341	Bolus	0.547		0.384	0.264	0.165	0.298		0.236	0.207
354	Bolus	0.567		0.098	0.054	0.081	0.480		0.090	0.176
390	Bolus	0.401		0.119	0.090	0.106	0.467		0.232	0.150
393	Bolus	0.180		0.037	0.039	0.204	0.328		0.143	0.121
396	Bolus	0.591		0.117	0.088	0.127	0.852		0.278	0.212
399	Bolus	0.141		0.077	0.045	0.111	0.431		0.089	0.107
436	Bolus	0.448		0.234	0.147	0.177	0.485		0.252	0.180
458	Bolus	0.221		0.050	0.055	0.250	0.511		0.340	0.101
519	Bolus	0.438		0.198	0.094	0.145	0.384		0.375	0.234
528	Bolus	0.496		0.078	0.039	0.048	0.339		0.259	0.074
529	Bolus	0.674		0.381	0.125	0.081	0.189		0.201	0.228
102	Bolus + Vit C	0.817		0.138	0.083	0.540	0.697		0.630	0.335
209	Bolus + Vit C	0.310		0.181	0.143	0.122	0.184		0.329	0.152
258	Bolus + Vit C	0.330		0.164	0.186	0.191	0.622		0.844	0.364
299	Bolus + Vit C	0.775		0.081	0.044	0.402	0.858		0.502	0.188
333	Bolus + Vit C	0.749		0.116	0.076	0.089	0.623		0.423	0.198
341	Bolus + Vit C	0.470		0.418	0.233	0.243	0.387		0.344	0.326
354	Bolus + Vit C	0.668		0.090	0.055	0.319	0.807		0.342	0.105
390	Bolus + Vit C	0.306		0.111	0.080	0.090	0.461		0.219	0.158
393	Bolus + Vit C	0.950		0.177	0.083	0.405	0.803		0.317	0.304
396	Bolus + Vit C	0.564		0.176	0.129	0.153	0.931		0.395	0.276
399	Bolus + Vit C	0.297		0.081	0.066	0.082	0.561		0.214	0.129
436	Bolus + Vit C	0.498		0.228	0.117	0.178	0.321		0.303	0.167
458	Bolus + Vit C	0.397		0.121	0.056	0.138	0.524		0.369	0.151
519	Bolus + Vit C	0.425		0.147	0.096	0.267	0.415		0.451	0.214
528	Bolus + Vit C	0.407		0.065	0.043	0.037	0.542		0.109	0.077
529	Bolus + Vit C	0.236		0.243	0.127	0.075	0.139		0.231	0.136
102	Sipping	0.587		0.163	0.107	0.195	0.672		0.563	0.264
209	Sipping	0.529		0.300	0.200	0.206	0.248		0.148	0.197
258	Sipping	0.604		0.504	0.221	0.440	0.347		0.159	0.296
299	Sipping	0.754		0.200	0.074	0.077	0.081		0.331	0.303
333	Sipping	0.485		0.137	0.080	0.040	0.127		0.373	0.088
341	Sipping	0.591		0.599	0.420	0.274	0.248		0.169	0.342
354	Sipping	0.899		0.209	0.088	0.144	0.296		0.178	0.144
390	Sipping	0.210		0.102	0.093	0.201	0.093		0.094	0.104
393	Sipping	0.283		0.124	0.088	0.222	0.108		0.092	0.090
396	Sipping	0.409		0.285	0.123	0.146	0.099		0.100	0.305
399	Sipping	0.376		0.083	0.062	0.078	0.384		0.190	0.179
436	Sipping	0.542		0.347	0.261	0.319	0.143		0.126	0.135
458	Sipping	0.402		0.097	0.060	0.074	0.166		0.475	0.172
519	Sipping	0.216		0.154	0.100	0.193	0.269		0.186	0.115
528	Sipping	0.247		0.223	0.080	0.058	0.038		0.046	0.093
529	Sipping	0.299		0.215	0.119	0.093	0.091		0.129	0.123
102	Sipping + Vit C	0.756		0.457	0.118	0.093	0.217		0.351	0.098
209	Sipping + Vit C	0.642		0.250	0.148	0.185	0.322		0.283	0.255
258	Sipping + Vit C	0.515		0.499	0.307	0.110	0.152		0.133	0.142
299	Sipping + Vit C	0.653		0.091	0.055	0.216	0.641		0.634	0.287
333	Sipping + Vit C	0.484		0.092	0.060	0.166	0.054		0.052	0.192
341	Sipping + Vit C	0.647		0.376	0.162	0.124	0.386		0.207	0.255
354	Sipping + Vit C	0.759		0.238	0.126	0.046	0.065		0.059	0.162
390	Sipping + Vit C	0.308		0.272	0.124	0.162	0.176		0.147	0.122
393	Sipping + Vit C	0.394		0.201	0.114	0.221	0.220		0.093	0.159
396	Sipping + Vit C	0.608		0.282	0.092	0.207	0.113		0.430	0.225
399	Sipping + Vit C	0.291		0.156	0.138	0.116	0.109		0.083	0.162
436	Sipping + Vit C	0.503		0.357	0.168	0.112	0.125		0.154	0.210
458	Sipping + Vit C	0.338		0.089	0.067	0.127	0.380		0.091	0.110
519	Sipping + Vit C	0.395		0.184	0.137	0.122	0.152		0.214	0.176
528	Sipping + Vit C	0.584		0.209	0.063	0.053	0.057		0.077	0.094
529	Sipping + Vit C	0.263		0.301	0.184	0.119	0.109		0.146	0.211

Supplementary Material:

Raw Data: Vitamin C (μmol/L)

ID	Treatment	0min	30min	60min	120min	180min	240min	270min	300min	360min
102	Bolus	20			17	21	21		22	17
209	Bolus	23			21	19	26		26	16
258	Bolus	14			11	9	13		14	13
299	Bolus	11			9	25	12		9	13
333	Bolus	9			6	8	11		8	8
341	Bolus	14			11	11	12		14	10
354	Bolus	10			8	8	7		7	6
390	Bolus	15			12	12	11		14	13
393	Bolus	17			16	16	15		20	17
396	Bolus	9			13	13	17		17	18
399	Bolus	25			20	22	19		21	18
436	Bolus	36			29	28	28		31	21
458	Bolus	35			36	32	36		36	24
519	Bolus	13			12	10	12		13	11
528	Bolus	14			18	18	11		14	11
529	Bolus	25			21	26	23		24	21
102	Bolus + Vit C	14			26	53	65		52	32
209	Bolus + Vit C	27			42	40	58		55	61
258	Bolus + Vit C	8			6	14	26		29	21
299	Bolus + Vit C	14			13	41	54		46	45
333	Bolus + Vit C	13			22	22	37		36	27
341	Bolus + Vit C	18			21	27	51		37	31
354	Bolus + Vit C	18			24	32	37		51	44
390	Bolus + Vit C	21			51	61	53		62	48
393	Bolus + Vit C	18			46	64	76		78	60
396	Bolus + Vit C	21			25	40	40		40	41
399	Bolus + Vit C	10			92	112	119		101	86
436	Bolus + Vit C	63			74	86	88		81	59
458	Bolus + Vit C	32			54	57	68		72	59
519	Bolus + Vit C	16			34	44	50		51	30
528	Bolus + Vit C	23			50	52	59		79	51
529	Bolus + Vit C	23			30	38	55		64	51
102	Sipping	17			18	20	19		18	15
209	Sipping	23			49	18	20		22	22
258	Sipping	5			5	5	5		5	5
299	Sipping	11			14	11	11		12	9
333	Sipping	8			8	7	13		10	7
341	Sipping	14			17	15	12		10	17
354	Sipping	9			12	9	9		12	7
390	Sipping	10			7	8	5		10	8
393	Sipping	18			19	17	18		18	19
396	Sipping	16			28	18	12		17	18
399	Sipping	13			12	14	14		16	15
436	Sipping	16			13	12	12		16	15
458	Sipping	31			29	29	26		6	23
519	Sipping	21			16	18	18		23	19
528	Sipping	29			29	29	36		37	30
529	Sipping	16			27	26	23		26	24
102	Sipping + Vit C	16			11	55	49		56	43
209	Sipping + Vit C	14			26	35	57		59	27
258	Sipping + Vit C	6			23	46	30		44	36
299	Sipping + Vit C	13			29	19	45		59	46
333	Sipping + Vit C	41			54	75	76		76	64
341	Sipping + Vit C	16			22	38	41		31	33
354	Sipping + Vit C	10			24	27	27		36	33
390	Sipping + Vit C	11			57	51	55		55	37
393	Sipping + Vit C	17			64	90	63		46	34
396	Sipping + Vit C	23			33	43	50		67	62
399	Sipping + Vit C	24			88	96	70		63	54
436	Sipping + Vit C	15			20	55	58		59	46
458	Sipping + Vit C	26			62	70	73		70	51
519	Sipping + Vit C	12			29	28	10		26	23
528	Sipping + Vit C	27			52	52	54		61	56
529	Sipping + Vit C	37			63	67	59		68	51

Supplementary Material:

Raw Data: TRAP (μmol/L)

ID	Treatment	0min	30min	60min	120min	180min	240min	270min	300min	360min
102	Bolus	1072			1027	1063	962		808	790
209	Bolus	1059			1140	1240	1038		1094	1038
258	Bolus	303			307	387	351		253	315
299	Bolus	1052			1020	1090	974		961	1070
333	Bolus	1035			1063	1192	1123		1037	1007
341	Bolus	1034			1264	1329	1278		1070	896
354	Bolus	1100			1285	1234	1158		1031	974
390	Bolus	1090			1133	1102	1146		1071	912
393	Bolus	1270			1710	1423	1334		905	1070
396	Bolus	1235			1267	1295	1216		1210	867
399	Bolus	1439			1368	1342	1105		1246	1080
436	Bolus	1497			1405	1398	1224		1340	1223
458	Bolus	1081			1269	1362	1400		1248	1044
519	Bolus	1076			1283	1282	1210		1100	1085
528	Bolus	1427			1828	1964	1742		1435	1133
529	Bolus	1197			1441	1406	1345		1345	1013
102	Bolus + Vit C	1113			1021	1059	972		1042	1094
209	Bolus + Vit C	1062			1170	1176	1090		1146	1075
258	Bolus + Vit C	283			268	298	263		290	450
299	Bolus + Vit C	993			1077	1085	1066		1112	1006
333	Bolus + Vit C	1182			1151	988	1094		1230	1078
341	Bolus + Vit C	1248			1309	1193	1404		1250	1221
354	Bolus + Vit C	1177			1237	1182	1254		1253	1345
390	Bolus + Vit C	1070			1086	1065	1084		1201	995
393	Bolus + Vit C	933			933	933	933		933	933
396	Bolus + Vit C	1127			1261	1134	1207		1206	1292
399	Bolus + Vit C	1147			1258	1347	1173		1294	1095
436	Bolus + Vit C	1261			1504	1476	1140		1537	1153
458	Bolus + Vit C	1066			1308	1471	1258		1127	1032
519	Bolus + Vit C	1223			1115	1338	1276		1154	1166
528	Bolus + Vit C	1559			1564	1715	1646		1728	1812
529	Bolus + Vit C	1316			1399	1462	1354		1185	1178
102	Sipping	1114			1166	1105	1003		910	929
209	Sipping	1120			1180	1153	1086		1079	1013
258	Sipping	497			539	533	512		511	515
299	Sipping	948			1042	1140	912		868	916
333	Sipping	995			1189	1128	992		1061	1102
341	Sipping	1153			1016	1268	1270		1237	1214
354	Sipping	1030			1102	1048	1104		992	1096
390	Sipping	1055			1108	1147	1071		1180	1053
393	Sipping	938			895	897	992		946	1025
396	Sipping	1089			1202	1198	1154		1099	1265
399	Sipping	1371			1281	1261	1155		1073	1034
436	Sipping	1250			1322	1259	1298		1312	1220
458	Sipping	917			983	925	1039		1054	907
519	Sipping	1140			1331	1277	1224		1155	1365
528	Sipping	1392			1520	1275	1580		1445	1372
529	Sipping	1046			1176	983	1113		1007	1154
102	Sipping + Vit C	1146			965	1041	989		932	923
209	Sipping + Vit C	1109			1234	1134	1096		1233	1280
258	Sipping + Vit C	499			538	559	543		532	608
299	Sipping + Vit C	969			1041	1076	905		988	1153
333	Sipping + Vit C	1091			1028	1194	1049		1006	1146
341	Sipping + Vit C	1133			1178	1352	1210		1207	1291
354	Sipping + Vit C	991			991	991	991		991	991
390	Sipping + Vit C	1023			1220	1055	1062		1113	1067
393	Sipping + Vit C	1034			1219	1146	1077		1186	1104
396	Sipping + Vit C	1012			1147	1166	1246		1311	1362
399	Sipping + Vit C	1089			1179	1178	1232		1120	1310
436	Sipping + Vit C	1223			1234	1263	1119		1341	1331
458	Sipping + Vit C	897			1016	1114	1088		1292	1127
519	Sipping + Vit C	1118			1216	1394	1237		1246	1241
528	Sipping + Vit C	1422			1321	1377	1417		1503	1589
529	Sipping + Vit C	1038			1044	1092	1031		1100	967

Supplementary Material:

Raw Data: Oxidized LDL (U/L)

ID	Treatment	0min	30min	60min	120min	180min	240min	270min	300min	360min
102	Bolus	81			91	115	113		79	69
209	Bolus	116			159	78	75		72	74
258	Bolus	116			53	83	62		38	46
299	Bolus	95			34	37	40		41	29
333	Bolus	51			45	49	43		37	44
341	Bolus	135			75	15	17		111	17
354	Bolus	16			44	41	59		69	53
390	Bolus	73			58	76	63		99	74
393	Bolus	132			42	58	66		91	101
396	Bolus	112			63	122	78		63	55
399	Bolus	64			154	96	64		109	48
436	Bolus	54			46	46	75		59	44
458	Bolus	42			38	62	58		58	61
519	Bolus	58			134	65	98		25	72
528	Bolus	24			56	26	29		35	33
529	Bolus	37			16	25	140		20	27
102	Bolus + Vit C	143			98	118	131		71	63
209	Bolus + Vit C	129			101	119	92		92	76
258	Bolus + Vit C	49			78	57	48		41	41
299	Bolus + Vit C	49			79	32	48		32	28
333	Bolus + Vit C	56			69	37	41		39	46
341	Bolus + Vit C	117			111	82	74		75	80
354	Bolus + Vit C	36			31	30	31		28	28
390	Bolus + Vit C	106			118	104	102		84	74
393	Bolus + Vit C	19			123	98	65		75	65
396	Bolus + Vit C	138			133	30	26		83	140
399	Bolus + Vit C	54			87	67	68		73	66
436	Bolus + Vit C	46			39	52	54		42	39
458	Bolus + Vit C	57			47	67	56		49	54
519	Bolus + Vit C	130			125	169	41		165	33
528	Bolus + Vit C	42			64	31	54		42	39
529	Bolus + Vit C	23			11	70	84		85	23
102	Sipping	84			85	141	31		149	123
209	Sipping	98			44	75	91		72	68
258	Sipping	94			133	120	50		42	126
299	Sipping	47			37	43	39		55	41
333	Sipping	42			29	44	42		52	48
341	Sipping	103			131	138	108		85	87
354	Sipping	27			28	31	40		33	30
390	Sipping	75			124	82	109		103	92
393	Sipping	77			86	100	74		71	122
396	Sipping	57			27	64	84		98	105
399	Sipping	69			71	74	63		59	66
436	Sipping	43			52	61	46		49	62
458	Sipping	63			40	45	44		36	53
519	Sipping	29			127	103	110		144	25
528	Sipping	41			35	36	37		26	39
529	Sipping	98			128	116	46		148	71
102	Sipping + Vit C	64			94	117	96		136	87
209	Sipping + Vit C	111			101	115	80		134	118
258	Sipping + Vit C	105			21	61	97		80	46
299	Sipping + Vit C	31			36	29	40		46	42
333	Sipping + Vit C	54			59	35	41		45	39
341	Sipping + Vit C	130			95	26	80		78	85
354	Sipping + Vit C	36			42	93	35		32	30
390	Sipping + Vit C	45			75	67	51		97	115
393	Sipping + Vit C	77			56	83	75		70	84
396	Sipping + Vit C	17			127	102	110		91	107
399	Sipping + Vit C	98			116	117	131		118	98
436	Sipping + Vit C	48			58	60	58		104	99
458	Sipping + Vit C	51			27	39	37		49	41
519	Sipping + Vit C	105			92	89	97		74	93
528	Sipping + Vit C	33			34	33	76		59	42
529	Sipping + Vit C	121			84	75	79		124	120

Supplementary Material:

Raw Data: CD/ApoB-100 (µmol)

ID	Treatment	0min	30min	60min	120min	180min	240min	270min	300min	360min
102	Bolus	16.1			20.7	13.0	18.3		20.4	26.6
209	Bolus	15.7			12.2	13.8	13.1		12.9	12.9
258	Bolus	13.8			19.2	15.8	16.9		4.4	16.2
299	Bolus	23.7			19.4	18.6	11.9		27.3	26.7
333	Bolus	18.0			34.4	23.5	16.3		15.9	22.8
341	Bolus	25.5			20.4	10.5	18.2		22.0	20.5
354	Bolus	14.9			21.5	2.1	17.7		5.4	12.4
390	Bolus	24.5			11.6	10.1	14.7		12.0	18.5
393	Bolus	17.0			17.0	26.3	22.1		18.6	20.1
396	Bolus	27.7			19.9	9.9	20.5		15.1	16.4
399	Bolus	13.3			28.4	12.1	31.9		50.6	15.6
436	Bolus	11.9			9.4	8.6	16.0		22.1	9.4
458	Bolus	15.0			11.6	10.7	7.4		16.2	12.3
519	Bolus	12.5			16.0	8.7	10.1		8.1	13.8
528	Bolus	8.4			6.9	6.2	5.7		5.9	7.1
529	Bolus	13.2			11.6	18.2	13.1		8.0	17.4
102	Bolus + Vit C	20.0			26.5	15.6	15.7		20.6	18.0
209	Bolus + Vit C	15.7			12.4	15.9	14.2		15.1	15.7
258	Bolus + Vit C	19.8			11.0	13.1	27.8		13.3	12.1
299	Bolus + Vit C	11.2			19.3	9.5	14.5		10.8	21.3
333	Bolus + Vit C	19.7			36.1	16.3	26.7		12.4	24.6
341	Bolus + Vit C	17.7			19.7	12.8	16.4		27.2	14.7
354	Bolus + Vit C	4.6			8.6	7.0	14.2		10.6	11.1
390	Bolus + Vit C	17.5			14.3	24.5	17.3		15.8	19.5
393	Bolus + Vit C	24.9			20.0	7.2	19.3		21.2	18.6
396	Bolus + Vit C	13.3			15.0	16.0	12.8		13.5	17.4
399	Bolus + Vit C	24.4			17.1	12.1	12.8		15.1	23.8
436	Bolus + Vit C	21.0			8.2	9.6	9.2		8.0	9.5
458	Bolus + Vit C	20.6			12.1	11.6	4.4		16.6	13.5
519	Bolus + Vit C	13.7			16.4	10.8	12.1		19.5	9.9
528	Bolus + Vit C	3.0			9.4	6.7	8.3		4.1	6.7
529	Bolus + Vit C	11.1			8.9	11.8	12.4		10.1	13.0
102	Sipping	14.1			30.3	21.3	20.3		24.8	34.2
209	Sipping	15.9			13.3	13.6	15.1		14.2	17.0
258	Sipping	19.2			13.3	15.4	13.4		12.0	10.0
299	Sipping	21.7			16.5	12.8	9.6		19.3	13.0
333	Sipping	22.9			11.0	16.8	43.5		7.3	4.8
341	Sipping	15.9			17.5	15.4	17.5		13.2	17.8
354	Sipping	24.9			11.4	4.0	2.2		10.4	15.9
390	Sipping	16.5			12.5	15.2	14.9		17.3	21.2
393	Sipping	19.1			26.6	29.2	22.5		48.7	27.3
396	Sipping	9.4			6.7	18.5	13.5		20.3	11.6
399	Sipping	18.0			22.8	19.4	26.8		18.7	29.9
436	Sipping	9.9			9.9	7.0	13.4		9.9	33.9
458	Sipping	10.1			12.2	9.4	16.3		11.4	17.4
519	Sipping	24.2			22.1	22.2	20.9		18.3	21.9
528	Sipping	4.6			4.7	8.7	3.1		4.3	6.4
529	Sipping	14.3			12.2	10.9	16.8		13.4	10.5
102	Sipping + Vit C	22.1			40.9	16.8	19.8		22.3	44.4
209	Sipping + Vit C	27.0			18.2	12.8	17.2		12.0	19.5
258	Sipping + Vit C	23.2			14.8	10.7	11.3		14.4	10.5
299	Sipping + Vit C	12.9			10.8	17.2	19.0		8.0	15.5
333	Sipping + Vit C	11.2			14.1	18.2	16.0		12.5	23.4
341	Sipping + Vit C	17.8			19.2	11.9	13.2		18.4	18.0
354	Sipping + Vit C	21.4			6.0	14.7	12.8		9.0	11.0
390	Sipping + Vit C	23.0			19.4	18.6	23.4		31.0	22.6
393	Sipping + Vit C	30.4			20.3	27.1	21.0		26.2	25.8
396	Sipping + Vit C	31.0			14.4	19.3	18.6		11.3	15.0
399	Sipping + Vit C	22.9			6.9	22.1	14.6		19.4	17.1
436	Sipping + Vit C	9.6			9.0	12.7	8.1		11.1	11.9
458	Sipping + Vit C	12.4			37.4	10.4	10.9		25.2	10.8
519	Sipping + Vit C	11.0			8.5	12.1	8.9		8.2	6.2
528	Sipping + Vit C	2.1			8.9	5.1	7.0		5.5	4.1
529	Sipping + Vit C	21.5			9.1	10.6	7.2		10.3	9.9

Supplementary Material:

Raw Data: Augmentation Index (%)

ID	Treatment	0min	30min	60min	120min	180min	240min	270min	300min	360min
102	Bolus	95.0		89.0	101.7		127.0		111.0	95.0
209	Bolus	61.5		37.0	34.5		39.5		32.5	44.5
258	Bolus	na		na	na		na		na	na
299	Bolus	74.0		58.0	65.5		76.0		58.0	55.0
333	Bolus	106.0		93.0	103.0		100.0		89.0	86.0
341	Bolus	79.0		78.0	80.0		87.0		69.0	69.5
354	Bolus	99.0		84.0	97.5		99.0		81.0	97.5
390	Bolus	55.5		54.0	57.5		52.5		53.0	53.0
393	Bolus	79.5		71.0	69.0		66.0		69.5	71.0
396	Bolus	55.0		53.5	51.8		48.5		39.5	48.0
399	Bolus	101.0		87.5	90.5		91.0		89.0	90.5
436	Bolus	74.5		63.5	74.0		68.0		59.5	71.0
458	Bolus	83.5		70.0	78.0		74.5		67.0	78.5
519	Bolus	92.5		91.5	89.0		81.5		75.0	81.0
528	Bolus	65.0		58.5	61.5		65.5		64.5	65.0
529	Bolus	87.5		85.5	84.0		98.0		68.0	71.0
102	Bolus + Vit C	88.5		89.5	97.0		94.5		84.0	84.5
209	Bolus + Vit C	68.0		53.5	66.5		65.0		51.0	61.0
258	Bolus + Vit C	na		na	na		na		na	na
299	Bolus + Vit C	72.5		53.0	72.5		71.0		46.0	59.5
333	Bolus + Vit C	92.5		84.0	94.5		89.5		80.5	87.0
341	Bolus + Vit C	68.5		70.0	71.0		71.5		53.0	61.0
354	Bolus + Vit C	80.5		69.0	83.0		93.5		69.0	92.5
390	Bolus + Vit C	54.0		49.5	47.0		51.0		48.5	46.0
393	Bolus + Vit C	52.5		63.5	61.3		57.0		50.5	59.0
396	Bolus + Vit C	58.0		52.5	48.5		43.5		36.0	39.0
399	Bolus + Vit C	116.5		100.5	90.0		93.5		101.5	94.0
436	Bolus + Vit C	74.5		63.5	65.5		68.5		62.0	64.5
458	Bolus + Vit C	61.0		68.5	72.5		68.5		52.5	60.0
519	Bolus + Vit C	97.5		90.0	97.5		84.0		76.5	77.5
528	Bolus + Vit C	81.5		68.0	72.0		67.0		70.0	72.5
529	Bolus + Vit C	101.5		115.0	94.0		91.0		76.0	75.0
102	Sipping	99.0		102.5	126.0		98.0		94.5	87.0
209	Sipping	76.0		77.5	69.5		67.0		61.5	65.0
258	Sipping	na		na	na		na		na	na
299	Sipping	67.5		64.0	62.0		56.0		50.0	62.0
333	Sipping	119.5		109.5	120.5		102.0		100.5	104.5
341	Sipping	70.5		66.5	72.5		77.5		64.5	66.0
354	Sipping	86.0		89.5	96.5		95.5		81.5	88.5
390	Sipping	64.5		56.0	60.0		56.0		59.0	60.5
393	Sipping	64.5		60.5	81.0		76.0		69.5	63.0
396	Sipping	51.5		49.5	53.0		48.0		35.0	42.0
399	Sipping	115.0		95.5	92.5		95.5		84.5	92.0
436	Sipping	69.0		78.0	83.5		81.0		73.0	81.5
458	Sipping	73.5		71.0	63.5		80.0		75.3	70.5
519	Sipping	95.5		98.0	93.5		90.0		84.5	85.0
528	Sipping	72.0		73.8	75.5		65.5		60.0	77.0
529	Sipping	86.0		89.5	83.0		88.0		84.0	81.0
102	Sipping + Vit C	102.5		97.5	102.2		111.5		88.0	99.0
209	Sipping + Vit C	79.0		68.0	70.5		62.5		56.0	64.0
258	Sipping + Vit C	na		na	na		na		na	na
299	Sipping + Vit C	63.5		65.5	66.0		70.0		53.0	60.0
333	Sipping + Vit C	99.0		106.3	113.5		101.0		85.0	96.0
341	Sipping + Vit C	69.0		68.0	66.5		74.5		69.0	63.5
354	Sipping + Vit C	102.0		94.5	98.5		105.0		83.0	93.5
390	Sipping + Vit C	63.0		53.0	58.5		51.5		44.5	58.0
393	Sipping + Vit C	74.0		81.0	78.0		75.0		63.0	65.0
396	Sipping + Vit C	68.0		51.0	60.5		61.0		48.5	48.0
399	Sipping + Vit C	100.0		100.3	100.5		108.5		103.0	88.0
436	Sipping + Vit C	74.5		70.5	77.0		82.0		65.5	73.5
458	Sipping + Vit C	71.0		76.5	79.0		84.5		71.0	69.5
519	Sipping + Vit C	102.0		94.7	94.3		93.5		89.0	96.5
528	Sipping + Vit C	68.5		61.0	70.0		65.5		53.5	61.0
529	Sipping + Vit C	94.0		97.0	92.5		93.5		82.5	80.0

Supplementary Material:

Raw Data: Augmentation Index normalized to a pulse pressure of 75mmHg (%)

ID	Treatment	0min	30min	60min	120min	180min	240min	270min	300min	360min
102	Bolus	91.0		88.0	100.3		125.0		110.8	96.5
209	Bolus	63.5		41.0	37.0		42.0		37.0	49.5
258	Bolus	na		na	na		na		na	na
299	Bolus	72.0		58.5	63.0		72.5		58.5	58.0
333	Bolus	101.5		91.5	101.3		95.0		87.5	84.5
341	Bolus	77.5		77.5	79.5		83.5		69.0	69.5
354	Bolus	97.0		84.5	96.0		98.5		82.0	97.0
390	Bolus	48.0		49.0	52.5		44.0		47.5	48.5
393	Bolus	73.5		68.0	66.0		62.0		65.5	67.0
396	Bolus	55.0		51.5	49.7		46.0		38.5	48.5
399	Bolus	99.0		87.0	89.5		89.0		91.0	90.0
436	Bolus	75.5		64.0	74.5		64.0		59.5	72.0
458	Bolus	83.5		70.0	76.5		73.5		65.5	79.5
519	Bolus	89.0		86.5	82.5		74.0		72.0	77.5
528	Bolus	64.0		59.5	61.5		63.0		65.5	66.5
529	Bolus	79.0		77.0	77.0		88.5		62.0	65.5
102	Bolus + Vit C	89.0		89.0	92.5		91.0		86.0	86.0
209	Bolus + Vit C	63.0		49.5	59.5		57.0		46.5	56.0
258	Bolus + Vit C	na		na	na		na		na	na
299	Bolus + Vit C	74.5		54.0	69.5		67.0		48.0	62.5
333	Bolus + Vit C	87.5		81.5	91.0		86.5		79.0	85.5
341	Bolus + Vit C	72.0		73.0	71.0		71.5		55.5	62.5
354	Bolus + Vit C	82.0		69.5	81.5		90.0		70.5	92.5
390	Bolus + Vit C	50.5		43.5	40.5		42.5		41.0	39.5
393	Bolus + Vit C	50.5		60.5	57.7		52.0		49.0	57.5
396	Bolus + Vit C	61.0		55.0	49.5		43.0		39.5	42.0
399	Bolus + Vit C	115.0		98.5	89.0		90.5		107.5	94.0
436	Bolus + Vit C	76.0		66.5	65.5		69.5		62.5	68.0
458	Bolus + Vit C	65.0		71.5	75.5		69.5		56.5	65.0
519	Bolus + Vit C	93.5		86.5	92.5		77.5		73.5	75.5
528	Bolus + Vit C	80.5		69.5	71.0		67.0		72.5	74.0
529	Bolus + Vit C	92.5		106.0	85.0		83.0		68.0	68.0
102	Sipping	100.5		102.5	120.0		94.5		92.5	87.5
209	Sipping	70.0		70.5	61.5		59.0		55.5	58.5
258	Sipping	na		na	na		na		na	na
299	Sipping	65.0		63.0	60.5		56.3		52.0	61.5
333	Sipping	115.5		105.0	116.5		97.5		100.5	103.5
341	Sipping	73.5		68.0	72.0		72.5		64.0	75.5
354	Sipping	86.5		87.0	96.5		94.5		83.5	90.0
390	Sipping	59.5		52.0	54.0		50.0		56.0	57.5
393	Sipping	60.0		55.0	74.5		70.0		64.0	58.0
396	Sipping	52.5		47.5	52.0		48.5		37.7	44.5
399	Sipping	114.0		94.5	92.0		94.0		86.5	93.0
436	Sipping	66.0		72.0	77.0		77.0		69.5	78.5
458	Sipping	75.0		69.5	62.5		80.0		76.5	73.0
519	Sipping	91.5		92.0	87.5		84.0		80.5	81.0
528	Sipping	71.0		72.3	73.5		64.5		59.0	76.5
529	Sipping	77.5		80.0	75.0		78.0		76.5	73.5
102	Sipping + Vit C	98.5		92.0	96.8		106.5		85.0	98.0
209	Sipping + Vit C	73.0		61.5	62.5		55.0		50.5	58.5
258	Sipping + Vit C	na		na	na		na		na	na
299	Sipping + Vit C	60.0		63.5	63.5		65.5		55.0	63.0
333	Sipping + Vit C	95.0		102.0	109.0		98.0		83.5	94.5
341	Sipping + Vit C	72.0		71.5	70.0		75.0		72.3	69.5
354	Sipping + Vit C	99.0		90.0	93.5		102.0		82.0	93.5
390	Sipping + Vit C	57.0		46.0	52.0		46.5		42.0	53.0
393	Sipping + Vit C	70.0		77.0	74.0		72.5		60.5	62.0
396	Sipping + Vit C	67.0		52.0	60.0		62.0		49.5	47.5
399	Sipping + Vit C	96.0		97.0	98.0		105.5		103.0	89.0
436	Sipping + Vit C	75.0		68.0	73.5		79.0		67.0	73.0
458	Sipping + Vit C	73.5		76.0	79.5		83.0		74.5	71.0
519	Sipping + Vit C	97.0		89.0	88.3		87.0		85.0	92.0
528	Sipping + Vit C	68.5		60.0	67.5		65.0		55.0	62.0
529	Sipping + Vit C	84.5		88.0	83.5		83.5		76.5	75.0

Supplementary Material:

Raw Data: Heart Rate (beats/min)

ID	Treatment	0min	30min	60min	120min	180min	240min	270min	300min	360min
102	Bolus	66.0		71.0	70.8		70.5		74.3	78.0
209	Bolus	80.5		84.5	81.0		81.0		86.5	86.0
258	Bolus	na		na	na		na		na	na
299	Bolus	71.5		77.0	69.0		67.0		76.0	81.5
333	Bolus	64.5		71.5	71.0		63.5		73.0	72.5
341	Bolus	72.0		74.5	73.5		67.5		76.0	75.0
354	Bolus	71.0		78.0	71.0		72.5		78.5	73.0
390	Bolus	58.5		62.0	63.5		56.0		62.5	64.5
393	Bolus	62.5		67.0	68.0		65.0		66.0	65.0
396	Bolus	75.0		71.5	70.8		69.5		72.5	75.0
399	Bolus	71.0		73.0	73.0		70.0		80.5	74.0
436	Bolus	77.5		76.0	74.5		66.0		75.0	77.5
458	Bolus	74.0		76.0	71.5		71.5		72.5	76.5
519	Bolus	66.5		64.0	60.5		58.0		68.0	66.5
528	Bolus	72.5		76.0	76.0		70.0		78.0	78.5
529	Bolus	55.5		56.0	59.0		53.5		61.5	62.0
102	Bolus + Vit C	76.5		74.5	65.5		66.5		80.0	79.0
209	Bolus + Vit C	63.0		64.5	59.0		57.0		64.5	63.5
258	Bolus + Vit C	na		na	na		na		na	na
299	Bolus + Vit C	78.5		77.5	69.0		66.0		80.0	81.0
333	Bolus + Vit C	63.5		69.0	66.5		68.0		72.5	71.5
341	Bolus + Vit C	84.5		82.0	76.0		74.5		80.0	78.5
354	Bolus + Vit C	79.0		76.5	72.0		67.0		78.0	75.0
390	Bolus + Vit C	67.0		60.5	59.5		54.5		57.5	60.5
393	Bolus + Vit C	70.5		68.5	66.5		62.5		71.0	72.0
396	Bolus + Vit C	82.5		81.0	77.0		73.5		82.5	80.5
399	Bolus + Vit C	71.5		70.0	73.0		68.5		87.5	74.0
436	Bolus + Vit C	78.0		82.0	75.0		76.5		75.0	82.0
458	Bolus + Vit C	84.0		82.0	81.5		77.0		85.0	85.5
519	Bolus + Vit C	66.0		66.5	62.5		59.5		68.5	70.0
528	Bolus + Vit C	74.0		77.5	73.0		75.0		81.0	79.5
529	Bolus + Vit C	53.0		54.0	55.0		55.0		57.0	59.0
102	Sipping	77.0		74.5	61.5		66.5		69.5	76.0
209	Sipping	61.0		58.5	57.0		56.5		61.5	59.5
258	Sipping	na		na	na		na		na	na
299	Sipping	70.0		71.0	71.0		75.5		80.0	73.5
333	Sipping	65.5		64.5	64.5		64.5		75.0	71.5
341	Sipping	81.5		78.0	75.0		64.0		73.0	98.0
354	Sipping	75.0		69.5	75.0		72.0		78.5	78.5
390	Sipping	63.5		65.0	60.5		62.0		67.0	66.5
393	Sipping	66.0		62.5	59.5		61.0		62.5	64.0
396	Sipping	77.0		71.0	74.0		76.0		81.3	81.5
399	Sipping	73.0		73.0	74.0		71.5		79.5	76.5
436	Sipping	68.0		61.0	60.0		65.0		66.5	68.5
458	Sipping	78.0		72.0	72.0		75.0		78.0	81.0
519	Sipping	64.5		61.0	60.5		61.0		66.0	66.5
528	Sipping	72.0		71.0	70.0		72.0		73.0	73.5
529	Sipping	54.5		52.5	57.5		51.0		57.0	56.5
102	Sipping + Vit C	65.0		62.0	62.5		63.5		73.0	72.0
209	Sipping + Vit C	61.5		60.0	56.0		58.0		61.5	62.5
258	Sipping + Vit C	na		na	na		na		na	na
299	Sipping + Vit C	68.0		70.0	70.0		65.5		80.0	81.5
333	Sipping + Vit C	65.5		65.0	64.5		68.0		72.5	72.0
341	Sipping + Vit C	82.0		83.5	83.5		76.0		82.8	89.5
354	Sipping + Vit C	68.5		64.5	64.0		68.0		73.0	76.0
390	Sipping + Vit C	61.0		58.5	60.0		63.5		69.5	63.0
393	Sipping + Vit C	65.0		66.5	66.0		68.0		69.5	68.0
396	Sipping + Vit C	72.0		78.0	74.5		76.0		77.5	73.5
399	Sipping + Vit C	66.0		67.8	69.5		68.0		75.0	76.0
436	Sipping + Vit C	69.0		68.5	67.0		69.0		76.5	73.0
458	Sipping + Vit C	81.5		75.0	76.0		72.0		83.0	78.5
519	Sipping + Vit C	62.5		61.3	61.4		61.5		66.0	63.5
528	Sipping + Vit C	74.5		74.0	69.5		72.0		78.5	76.5
529	Sipping + Vit C	53.0		53.5	52.5		52.0		61.5	62.5

Supplementary Material:

Raw Data: Systolic Blood Pressure (mmHg)

ID	Treatment	0min	30min	60min	120min	180min	240min	270min	300min	360min
102	Bolus	140		150	144		155		161	166
209	Bolus	120		128	126		119		126	126
258	Bolus	88		103	99		100		106	101
299	Bolus	124		134	128		117		112	120
333	Bolus	143		137	128		136		127	122
341	Bolus	130		133	133		124		127	143
354	Bolus	124		120	111		125		115	104
390	Bolus	119		126	122		115		128	133
393	Bolus	126		135	141		134		135	138
396	Bolus	113		115	119		126		134	122
399	Bolus	131		156	134		140		143	147
436	Bolus	123		116	109		100		115	117
458	Bolus	130		137	125		128		130	127
519	Bolus	134		134	121		125		120	123
528	Bolus	104		101	101		95		97	102
529	Bolus	120		120	113		122		107	113
102	Bolus + Vit C	141		149	152		138		142	143
209	Bolus + Vit C	110		115	113		115		119	117
258	Bolus + Vit C	121		135	118		108		119	113
299	Bolus + Vit C	125		131	117		130		129	131
333	Bolus + Vit C	136		130	133		120		114	121
341	Bolus + Vit C	141		133	135		130		144	147
354	Bolus + Vit C	118		111	109		117		101	114
390	Bolus + Vit C	115		133	138		128		138	147
393	Bolus + Vit C	143		133	135		138		136	140
396	Bolus + Vit C	113		108	119		113		118	120
399	Bolus + Vit C	140		144	142		154		148	161
436	Bolus + Vit C	121		124	115		111		121	120
458	Bolus + Vit C	122		130	130		129		128	124
519	Bolus + Vit C	139		131	123		116		124	122
528	Bolus + Vit C	103		95	98		92		95	94
529	Bolus + Vit C	128		110	115		113		102	110
102	Sipping	135		137	145		148		146	131
209	Sipping	122		133	132		121		124	123
258	Sipping	102		103	98		112		100	99
299	Sipping	118		119	118		112		119	113
333	Sipping	119		162	136		138		160	141
341	Sipping	124		120	122		122		121	130
354	Sipping	120		115	114		113		108	100
390	Sipping	134		131	121		134		136	148
393	Sipping	129		134	136		134		135	135
396	Sipping	118		111	117		125		125	131
399	Sipping	141		139	144		145		142	127
436	Sipping	109		102	115		104		107	104
458	Sipping	112		117	117		117		116	115
519	Sipping	130		120	112		126		117	116
528	Sipping	95		95	96		92		93	95
529	Sipping	125		112	119		120		115	122
102	Sipping + Vit C	153		160	147		121		151	154
209	Sipping + Vit C	115		112	120		109		120	119
258	Sipping + Vit C	107		103	102		101		111	102
299	Sipping + Vit C	122		122	114		117		123	122
333	Sipping + Vit C	147		134	121		137		123	129
341	Sipping + Vit C	137		128	128		131		134	136
354	Sipping + Vit C	109		112	108		115		111	109
390	Sipping + Vit C	127		133	132		128		134	134
393	Sipping + Vit C	127		140	139		142		135	139
396	Sipping + Vit C	115		109	112		109		116	110
399	Sipping + Vit C	133		128	122		130		117	121
436	Sipping + Vit C	125		103	117		126		127	132
458	Sipping + Vit C	119		136	115		127		128	143
519	Sipping + Vit C	135		124	126		129		129	121
528	Sipping + Vit C	100		98	97		95		92	98
529	Sipping + Vit C	116		106	102		116		104	110

Supplementary Material:

Raw Data: Diastolic Blood Pressure (mmHg)

ID	Treatment	0min	30min	60min	120min	180min	240min	270min	300min	360min
102	Bolus	92		88	98		100		99	97
209	Bolus	72		75	72		74		71	75
258	Bolus	52		61	50		57		60	58
299	Bolus	70		71	70		70		64	75
333	Bolus	93		76	78		87		79	79
341	Bolus	89		84	87		92		80	91
354	Bolus	81		73	73		79		77	75
390	Bolus	77		78	74		73		82	76
393	Bolus	82		78	91		88		78	77
396	Bolus	70		73	70		63		73	67
399	Bolus	84		99	89		89		85	97
436	Bolus	66		66	73		65		64	68
458	Bolus	76		81	74		78		73	69
519	Bolus	73		73	70		73		71	67
528	Bolus	62		59	61		49		57	56
529	Bolus	70		69	62		69		55	62
102	Bolus + Vit C	93		90	99		93		89	91
209	Bolus + Vit C	63		63	71		67		64	66
258	Bolus + Vit C	63		79	66		61		68	71
299	Bolus + Vit C	74		74	76		78		69	75
333	Bolus + Vit C	82		73	77		74		75	77
341	Bolus + Vit C	98		74	87		81		88	89
354	Bolus + Vit C	83		72	72		76		66	74
390	Bolus + Vit C	75		75	77		72		79	85
393	Bolus + Vit C	79		75	80		89		67	79
396	Bolus + Vit C	74		74	80		70		70	71
399	Bolus + Vit C	82		89	93		102		89	105
436	Bolus + Vit C	79		72	74		74		72	71
458	Bolus + Vit C	77		75	75		75		75	76
519	Bolus + Vit C	82		75	75		68		68	68
528	Bolus + Vit C	60		54	54		55		54	56
529	Bolus + Vit C	75		66	66		67		55	63
102	Sipping	101		88	103		93		95	96
209	Sipping	66		61	64		63		61	63
258	Sipping	53		53	53		67		54	49
299	Sipping	70		74	66		70		70	69
333	Sipping	77		98	84		90		102	83
341	Sipping	82		78	83		80		79	84
354	Sipping	84		78	76		73		71	67
390	Sipping	80		78	76		82		85	85
393	Sipping	83		83	87		88		84	80
396	Sipping	72		64	71		67		66	67
399	Sipping	93		87	93		89		86	82
436	Sipping	68		61	72		65		63	62
458	Sipping	59		72	74		71		70	69
519	Sipping	82		71	65		71		60	64
528	Sipping	57		53	55		58		53	58
529	Sipping	68		65	70		70		67	72
102	Sipping + Vit C	100		92	90		86		98	94
209	Sipping + Vit C	63		64	70		66		67	69
258	Sipping + Vit C	65		59	59		60		63	58
299	Sipping + Vit C	71		70	65		68		59	66
333	Sipping + Vit C	87		81	74		84		71	72
341	Sipping + Vit C	88		87	85		84		83	82
354	Sipping + Vit C	71		70	74		74		70	72
390	Sipping + Vit C	79		65	78		82		80	82
393	Sipping + Vit C	81		89	90		84		73	86
396	Sipping + Vit C	67		65	67		67		64	65
399	Sipping + Vit C	87		84	80		84		70	68
436	Sipping + Vit C	70		63	64		67		58	72
458	Sipping + Vit C	81		88	71		73		73	81
519	Sipping + Vit C	79		71	68		74		69	65
528	Sipping + Vit C	59		54	57		54		55	57
529	Sipping + Vit C	68		62	59		63		51	63

Supplementary Material:

Raw Data: Pulse Pressure (mmHg)

ID	Treatment	0min	30min	60min	120min	180min	240min	270min	300min	360min
102	Bolus	48		62	60		55		62	69
209	Bolus	48		53	54		45		55	51
258	Bolus	36		42	49		43		46	43
299	Bolus	54		63	58		47		48	45
333	Bolus	50		50	50		49		49	43
341	Bolus	41		49	46		32		47	52
354	Bolus	43		47	38		46		38	29
390	Bolus	42		48	48		42		46	57
393	Bolus	44		57	50		46		57	61
396	Bolus	43		42	48		55		61	55
399	Bolus	47		57	45		51		58	56
436	Bolus	57		50	36		35		51	49
458	Bolus	54		56	51		50		57	58
519	Bolus	61		61	51		52		49	56
528	Bolus	42		42	40		46		40	46
529	Bolus	50		51	51		53		52	51
102	Bolus + Vit C	48		59	53		45		53	52
209	Bolus + Vit C	47		52	42		48		55	51
258	Bolus + Vit C	42		47	52		47		51	42
299	Bolus + Vit C	51		57	41		52		60	56
333	Bolus + Vit C	54		57	56		46		39	44
341	Bolus + Vit C	43		59	48		49		56	58
354	Bolus + Vit C	35		39	37		41		35	40
390	Bolus + Vit C	40		58	61		56		59	62
393	Bolus + Vit C	64		59	54		49		69	61
396	Bolus + Vit C	39		34	39		43		48	49
399	Bolus + Vit C	58		55	54		52		59	56
436	Bolus + Vit C	42		52	41		37		49	49
458	Bolus + Vit C	45		55	55		54		53	48
519	Bolus + Vit C	57		56	48		48		56	54
528	Bolus + Vit C	43		41	44		37		41	38
529	Bolus + Vit C	53		44	49		46		47	47
102	Sipping	34		49	42		55		51	35
209	Sipping	56		72	68		58		63	60
258	Sipping	49		50	45		45		46	50
299	Sipping	48		45	52		42		49	44
333	Sipping	42		64	52		48		58	58
341	Sipping	42		42	39		42		42	46
354	Sipping	36		37	38		40		37	33
390	Sipping	54		53	45		52		51	63
393	Sipping	46		51	49		46		51	55
396	Sipping	46		47	46		58		59	64
399	Sipping	48		52	51		56		56	45
436	Sipping	41		41	43		39		44	42
458	Sipping	53		45	43		46		46	46
519	Sipping	48		49	47		55		57	52
528	Sipping	38		42	41		34		40	37
529	Sipping	57		47	49		50		48	50
102	Sipping + Vit C	53		68	57		35		53	60
209	Sipping + Vit C	52		48	50		43		53	50
258	Sipping + Vit C	42		44	43		41		48	44
299	Sipping + Vit C	51		52	49		49		64	56
333	Sipping + Vit C	60		53	47		53		53	57
341	Sipping + Vit C	49		41	43		47		51	54
354	Sipping + Vit C	38		42	34		41		41	37
390	Sipping + Vit C	48		68	54		46		54	52
393	Sipping + Vit C	46		51	49		58		62	53
396	Sipping + Vit C	48		44	45		42		52	45
399	Sipping + Vit C	46		44	42		46		47	53
436	Sipping + Vit C	55		40	53		59		69	60
458	Sipping + Vit C	38		41	44		54		55	62
519	Sipping + Vit C	56		53	58		55		60	56
528	Sipping + Vit C	41		44	40		41		37	41
529	Sipping + Vit C	48		46	43		53		53	47

Supplementary Material:

Raw Data: Cholesterol (mmol/L)

ID	Treatment	0min	30min	60min	120min	180min	240min	270min	300min	360min
102	Bolus	6.46			6.45		6.59		6.62	6.46
209	Bolus	5.00			4.98		4.92		5.02	5.07
258	Bolus	5.47			5.54		5.39		5.22	5.39
299	Bolus	5.93			5.84		5.80		5.77	5.90
333	Bolus	3.85			3.57		3.79		3.59	3.63
341	Bolus	5.42			5.06		5.31		5.27	5.21
354	Bolus	5.14			4.90		5.03		4.92	4.97
390	Bolus	5.92			5.81		6.07		5.69	5.77
393	Bolus	4.88			4.76		4.78		4.58	4.83
396	Bolus	5.17			5.10		5.25		5.09	5.34
399	Bolus	4.57			4.38		4.44		4.32	4.40
436	Bolus	5.32			5.33		5.38		5.07	5.32
458	Bolus	3.63			3.68		3.71		3.61	3.62
519	Bolus	5.39			5.00		5.44		5.17	5.14
528	Bolus	5.73			5.78		5.99		5.96	5.77
529	Bolus	5.20			5.16		5.39		5.20	5.22
102	Bolus + Vit C	5.09			4.77		4.78		4.88	4.90
209	Bolus + Vit C	5.76			5.71		5.50		5.53	5.67
258	Bolus + Vit C	3.67			3.51		3.47		3.39	3.50
299	Bolus + Vit C	5.73			5.52		5.49		5.35	5.54
333	Bolus + Vit C	5.38			5.21		5.08		4.87	4.52
341	Bolus + Vit C	6.26			6.28		6.38		6.59	6.06
354	Bolus + Vit C	5.18			4.79		4.02		4.58	4.64
390	Bolus + Vit C	4.91			4.87		5.00		4.82	4.86
393	Bolus + Vit C	4.91			4.45		4.55		4.52	4.42
396	Bolus + Vit C	6.53			5.88		5.81		5.96	6.11
399	Bolus + Vit C	3.75			3.61		3.60		3.35	3.37
436	Bolus + Vit C	6.64			5.55		5.42		5.30	5.23
458	Bolus + Vit C	6.79			6.76		6.83		6.14	6.05
519	Bolus + Vit C	5.07			4.92		4.92		4.97	5.06
528	Bolus + Vit C	5.65			5.27		5.61		5.83	5.74
529	Bolus + Vit C	5.80			5.80		5.60		5.70	5.83
102	Sipping	3.81			3.50		3.53		3.53	3.65
209	Sipping	5.25			5.26		5.45		5.47	5.54
258	Sipping	5.69			5.29		5.27		5.02	5.26
299	Sipping	5.46			5.26		5.28		5.47	5.55
333	Sipping	5.22			5.00		5.01		4.96	5.10
341	Sipping	5.20			5.23		5.05		5.25	5.15
354	Sipping	4.60			4.33		4.49		4.66	4.53
390	Sipping	6.38			5.83		6.06		6.13	6.25
393	Sipping	3.44			3.31		3.23		3.10	3.16
396	Sipping	5.16			4.79		4.80		4.89	5.04
399	Sipping	6.60			6.52		6.31		6.60	6.28
436	Sipping	5.45			5.06		5.04		5.11	5.25
458	Sipping	5.59			5.41		5.51		5.54	5.70
519	Sipping	6.46			6.51		6.47		6.19	6.53
528	Sipping	3.75			3.51		3.44		3.62	3.56
529	Sipping	5.78			5.59		5.69		5.34	5.69
102	Sipping + Vit C	4.89			4.83		4.98		4.80	4.93
209	Sipping + Vit C	6.00			5.99		6.08		6.08	6.08
258	Sipping + Vit C	5.18			5.02		4.89		4.94	5.27
299	Sipping + Vit C	5.45			5.66		5.60		5.66	5.78
333	Sipping + Vit C	4.45			4.25		4.20		4.15	4.10
341	Sipping + Vit C	5.35			5.29		5.28		5.10	5.29
354	Sipping + Vit C	3.31			3.27		3.25		3.22	3.23
390	Sipping + Vit C	4.95			4.68		4.86		4.66	5.04
393	Sipping + Vit C	6.46			6.45		6.59		6.62	6.46
396	Sipping + Vit C	5.00			4.98		4.92		5.02	5.07
399	Sipping + Vit C	5.47			5.54		5.39		5.22	5.39
436	Sipping + Vit C	5.93			5.84		5.80		5.77	5.90
458	Sipping + Vit C	3.85			3.57		3.79		3.59	3.63
519	Sipping + Vit C	5.42			5.06		5.31		5.27	5.21
528	Sipping + Vit C	5.14			4.90		5.03		4.92	4.97
529	Sipping + Vit C	5.92			5.81		6.07		5.69	5.77

Supplementary Material:

Raw Data: Triglycerides (mmol/L)

ID	Treatment	0min	30min	60min	120min	180min	240min	270min	300min	360min
102	Bolus	1.50			1.27		1.31		1.37	1.81
209	Bolus	2.44			2.57		2.56		2.58	2.71
258	Bolus	1.03			0.90		1.05		1.12	1.15
299	Bolus	3.28			2.56		1.65		1.62	2.32
333	Bolus	0.72			0.57		0.68		0.72	1.03
341	Bolus	1.28			1.24		1.34		1.44	1.88
354	Bolus	0.83			0.75		1.01		1.13	1.56
390	Bolus	0.91			0.96		1.25		1.11	1.50
393	Bolus	0.74			0.84		0.89		0.94	1.25
396	Bolus	1.32			1.43		1.43		1.44	1.83
399	Bolus	0.84			0.83		0.92		0.94	1.10
436	Bolus	1.41			1.40		1.31		1.48	2.12
458	Bolus	0.77			0.58		0.59		0.61	0.74
519	Bolus	2.22			1.73		1.78		1.79	2.41
528	Bolus	1.89			1.66		1.44		1.47	2.14
529	Bolus	2.30			2.53		2.55		2.72	3.07
102	Bolus + Vit C	0.85			0.66		0.77		0.88	0.98
209	Bolus + Vit C	3.12			2.13		1.31		1.68	2.23
258	Bolus + Vit C	0.75			0.59		0.69		0.65	0.84
299	Bolus + Vit C	1.76			1.72		1.82		1.90	2.45
333	Bolus + Vit C	0.64			0.38		0.69		0.96	1.21
341	Bolus + Vit C	0.96			1.12		1.21		1.24	1.32
354	Bolus + Vit C	1.67			1.58		1.42		1.62	2.56
390	Bolus + Vit C	1.59			1.66		1.82		1.81	2.00
393	Bolus + Vit C	0.86			0.68		0.67		0.80	1.13
396	Bolus + Vit C	2.60			2.28		2.04		2.23	2.61
399	Bolus + Vit C	0.51			0.39		0.35		0.37	0.59
436	Bolus + Vit C	1.67			1.39		1.21		1.20	1.65
458	Bolus + Vit C	1.23			1.31		1.24		1.17	2.22
519	Bolus + Vit C	2.07			2.20		2.27		2.31	2.65
528	Bolus + Vit C	0.81			0.78		0.86		0.98	1.43
529	Bolus + Vit C	3.31			3.51		1.80		1.86	2.41
102	Sipping	0.76			0.56		0.61		0.71	1.18
209	Sipping	1.41			1.67		1.79		1.80	2.24
258	Sipping	1.03			0.75		0.96		0.95	1.71
299	Sipping	1.18			1.29		1.32		1.40	2.40
333	Sipping	1.11			1.12		1.22		1.27	1.72
341	Sipping	1.59			1.58		1.61		1.70	1.97
354	Sipping	0.71			0.59		0.57		0.75	1.01
390	Sipping	2.83			2.66		2.69		2.76	3.70
393	Sipping	0.49			0.40		0.33		0.32	0.92
396	Sipping	1.59			1.70		0.48		1.82	2.54
399	Sipping	1.95			1.81		1.54		1.68	2.58
436	Sipping	2.28			1.77		1.57		1.86	2.39
458	Sipping	0.85			0.70		0.80		0.81	1.36
519	Sipping	2.37			1.99		1.70		1.70	2.62
528	Sipping	0.59			0.47		0.43		0.49	0.99
529	Sipping	1.81			1.76		1.65		1.57	2.17
102	Sipping + Vit C	0.68			0.58		0.78		0.88	1.83
209	Sipping + Vit C	1.02			1.00		0.94		0.98	1.43
258	Sipping + Vit C	1.02			0.93		0.93		1.04	1.96
299	Sipping + Vit C	1.93			1.95		1.90		2.11	2.63
333	Sipping + Vit C	0.75			0.59		0.63		0.76	0.97
341	Sipping + Vit C	1.26			1.28		1.19		1.25	2.37
354	Sipping + Vit C	0.51			0.34		0.27		0.26	0.69
390	Sipping + Vit C	1.06			1.08		1.10		1.12	1.77
393	Sipping + Vit C	1.50			1.27		1.31		1.37	1.81
396	Sipping + Vit C	2.44			2.57		2.56		2.58	2.71
399	Sipping + Vit C	1.03			0.90		1.05		1.12	1.15
436	Sipping + Vit C	3.28			2.56		1.65		1.62	2.32
458	Sipping + Vit C	0.72			0.57		0.68		0.72	1.03
519	Sipping + Vit C	1.28			1.24		1.34		1.44	1.88
528	Sipping + Vit C	0.83			0.75		1.01		1.13	1.56
529	Sipping + Vit C	0.91			0.96		1.25		1.11	1.50

Supplementary Material:

Raw Data: HDL-cholesterol (mmol/L)

ID	Treatment	0min	30min	60min	120min	180min	240min	270min	300min	360min
102	Bolus	1.26			1.29		1.31		1.31	1.23
209	Bolus	1.32			1.27		1.27		1.29	1.27
258	Bolus	1.69			1.70		1.69		1.64	1.65
299	Bolus	0.97			1.06		1.20		1.20	1.19
333	Bolus	1.58			1.56		1.64		1.54	1.53
341	Bolus	1.02			0.99		1.03		1.00	0.95
354	Bolus	1.66			1.64		1.66		1.59	1.56
390	Bolus	1.15			1.13		1.17		1.10	1.05
393	Bolus	1.47			1.44		1.48		1.42	1.42
396	Bolus	1.25			1.22		1.28		1.23	1.23
399	Bolus	1.29			1.23		1.22		1.18	1.17
436	Bolus	1.27			1.23		1.25		1.20	1.19
458	Bolus	1.64			1.71		1.77		1.70	1.68
519	Bolus	0.94			0.88		0.92		0.90	0.87
528	Bolus	1.09			1.11		1.15		1.16	1.11
529	Bolus	1.35			1.31		1.33		1.08	1.26
102	Bolus + Vit C	1.71			1.66		1.69		1.70	1.67
209	Bolus + Vit C	0.97			1.02		1.10		1.11	1.11
258	Bolus + Vit C	1.50			1.47		1.49		1.46	1.46
299	Bolus + Vit C	1.15			1.17		1.17		1.12	1.13
333	Bolus + Vit C	1.74			1.81		1.79		1.73	1.58
341	Bolus + Vit C	1.03			1.01		1.03		1.04	0.93
354	Bolus + Vit C	1.09			1.08		0.91		1.01	0.97
390	Bolus + Vit C	1.25			1.24		1.27		1.21	1.18
393	Bolus + Vit C	1.36			1.30		1.35		1.31	1.25
396	Bolus + Vit C	1.22			1.15		1.14		1.17	1.17
399	Bolus + Vit C	1.66			1.67		1.71		1.64	1.56
436	Bolus + Vit C	1.20			1.04		1.04		1.00	0.94
458	Bolus + Vit C	1.30			1.30		1.33		1.21	1.16
519	Bolus + Vit C	1.18			1.13		1.13		1.16	1.14
528	Bolus + Vit C	1.73			1.67		1.77		1.83	1.75
529	Bolus + Vit C	1.10			1.06		1.14		1.19	1.19
102	Sipping	1.47			1.41		1.46		1.47	1.43
209	Sipping	1.15			1.15		1.16		1.15	1.13
258	Sipping	1.62			1.59		1.60		1.54	1.52
299	Sipping	1.05			1.03		1.00		1.00	0.99
333	Sipping	1.25			1.21		1.24		1.19	1.16
341	Sipping	1.26			1.23		1.20		1.22	1.18
354	Sipping	1.37			1.31		1.36		1.40	1.29
390	Sipping	1.19			1.11		1.12		1.15	1.14
393	Sipping	1.53			1.51		1.56		1.51	1.49
396	Sipping	1.00			0.92		0.91		0.93	0.90
399	Sipping	1.13			1.15		1.13		1.17	1.10
436	Sipping	1.31			1.32		1.35		1.37	1.33
458	Sipping	1.66			1.65		1.67		1.64	1.64
519	Sipping	1.13			1.18		1.20		1.16	1.18
528	Sipping	1.55			1.47		1.49		1.55	1.52
529	Sipping	1.06			1.03		1.07		1.00	1.00
102	Sipping + Vit C	1.59			1.66		1.69		1.61	1.62
209	Sipping + Vit C	1.04			1.06		1.04		1.06	1.02
258	Sipping + Vit C	1.39			1.37		1.35		1.33	1.32
299	Sipping + Vit C	1.23			1.27		1.21		1.23	1.20
333	Sipping + Vit C	1.14			1.22		1.22		1.16	1.14
341	Sipping + Vit C	1.18			1.17		1.18		1.14	1.15
354	Sipping + Vit C	1.41			1.44		1.50		1.51	1.49
390	Sipping + Vit C	1.03			1.00		1.04		0.98	0.99
393	Sipping + Vit C	1.26			1.29		1.31		1.31	1.23
396	Sipping + Vit C	1.32			1.27		1.27		1.29	1.27
399	Sipping + Vit C	1.69			1.70		1.69		1.64	1.65
436	Sipping + Vit C	0.97			1.06		1.20		1.20	1.19
458	Sipping + Vit C	1.58			1.56		1.64		1.54	1.53
519	Sipping + Vit C	1.02			0.99		1.03		1.00	0.95
528	Sipping + Vit C	1.66			1.64		1.66		1.59	1.56
529	Sipping + Vit C	1.15			1.13		1.17		1.10	1.05

Supplementary Material:

Raw Data: LDL-cholesterol (mmol/L)

ID	Treatment	0min	30min	60min	120min	180min	240min	270min	300min	360min
102	Bolus	4.52			4.58		4.68		4.69	4.41
209	Bolus	2.57			2.54		2.49		2.56	2.57
258	Bolus	3.31			3.43		3.22		3.07	3.22
299	Bolus	3.47			3.62		3.85		3.83	3.66
333	Bolus	1.94			1.75		1.84		1.72	1.63
341	Bolus	3.82			3.51		3.67		3.62	3.41
354	Bolus	3.10			2.92		2.91		2.82	2.70
390	Bolus	4.36			4.24		4.33		4.09	4.04
393	Bolus	3.07			2.94		2.90		2.73	2.84
396	Bolus	3.32			3.23		3.32		3.21	3.28
399	Bolus	2.90			2.77		2.80		2.71	2.73
436	Bolus	3.41			3.46		3.53		3.20	3.17
458	Bolus	1.64			1.71		1.67		1.63	1.60
519	Bolus	3.44			3.33		3.71		3.46	3.17
528	Bolus	3.78			3.92		4.19		4.13	3.69
529	Bolus	2.80			2.70		2.90		2.88	2.56
102	Bolus + Vit C	2.99			2.81		2.74		2.78	2.78
209	Bolus + Vit C	3.37			3.72		3.80		3.66	3.55
258	Bolus + Vit C	1.83			1.77		1.67		1.63	1.66
299	Bolus + Vit C	3.78			3.57		3.49		3.37	3.30
333	Bolus + Vit C	3.35			3.23		2.98		2.70	2.39
341	Bolus + Vit C	4.79			4.76		4.80		4.99	4.53
354	Bolus + Vit C	3.33			2.99		2.46		2.83	2.51
390	Bolus + Vit C	2.94			2.88		2.90		2.79	2.77
393	Bolus + Vit C	3.16			2.84		2.90		2.85	2.66
396	Bolus + Vit C	4.13			3.69		3.74		3.78	3.75
399	Bolus + Vit C	1.86			1.76		1.73		1.54	1.54
436	Bolus + Vit C	4.68			3.88		3.83		3.75	3.54
458	Bolus + Vit C	4.93			4.86		4.94		4.40	3.88
519	Bolus + Vit C	2.95			2.79		2.76		2.76	2.72
528	Bolus + Vit C	3.55			3.25		3.45		3.55	3.34
529	Bolus + Vit C	3.20			3.14		3.64		3.66	3.54
102	Sipping	1.99			1.84		1.79		1.74	1.68
209	Sipping	3.46			3.35		3.48		3.50	3.39
258	Sipping	3.60			3.36		3.23		3.05	2.96
299	Sipping	3.87			3.64		3.68		3.83	3.47
333	Sipping	3.47			3.28		3.22		3.19	3.16
341	Sipping	3.22			3.28		3.12		3.26	3.07
354	Sipping	2.91			2.75		2.87		2.92	2.78
390	Sipping	3.90			3.51		3.72		3.73	3.43
393	Sipping	1.69			1.62		1.52		1.44	1.25
396	Sipping	3.44			3.10		3.67		3.13	2.99
399	Sipping	4.58			4.55		4.48		4.67	4.01
436	Sipping	3.10			2.94		2.98		2.89	2.83
458	Sipping	3.54			3.44		3.48		3.53	3.44
519	Sipping	4.25			4.43		4.50		4.26	4.16
528	Sipping	1.93			1.83		1.75		1.85	1.59
529	Sipping	3.90			3.76		3.87		3.63	3.70
102	Sipping + Vit C	2.99			2.91		2.94		2.79	2.48
209	Sipping + Vit C	4.50			4.48		4.61		4.57	4.41
258	Sipping + Vit C	3.33			3.23		3.12		3.14	3.06
299	Sipping + Vit C	3.34			3.50		3.53		3.47	3.38
333	Sipping + Vit C	2.97			2.76		2.69		2.64	2.52
341	Sipping + Vit C	3.60			3.54		3.56		3.39	3.06
354	Sipping + Vit C	1.67			1.68		1.63		1.59	1.43
390	Sipping + Vit C	3.44			3.19		3.32		3.17	3.25
393	Sipping + Vit C	4.52			4.58		4.68		4.69	4.41
396	Sipping + Vit C	2.57			2.54		2.49		2.56	2.57
399	Sipping + Vit C	3.31			3.43		3.22		3.07	3.22
436	Sipping + Vit C	3.47			3.62		3.85		3.83	3.66
458	Sipping + Vit C	1.94			1.75		1.84		1.72	1.63
519	Sipping + Vit C	3.82			3.51		3.67		3.62	3.41
528	Sipping + Vit C	3.10			2.92		2.91		2.82	2.70
529	Sipping + Vit C	4.36			4.24		4.33		4.09	4.04

Supplementary Material:

Raw Data: C-reactive protein (mg/L)

ID	Treatment	0min	30min	60min	120min	180min	240min	270min	300min	360min
102	Bolus	4.3			1.9		3.9		1.7	4.1
209	Bolus	9.4			8.7		8.9		8.1	8.4
258	Bolus	5.1			5.9		6.4		6.5	6.6
299	Bolus	3.9			3.7		3.8		3.9	1.7
333	Bolus	11.7			11.0		11.3		11.2	12.0
341	Bolus	0.3			0.3		0.4		0.4	0.4
354	Bolus	0.7			0.6		0.6		0.6	0.6
390	Bolus	0.5			0.4		0.4		0.4	0.3
393	Bolus	0.3			0.3		0.3		0.3	0.3
396	Bolus	7.8			6.7		7.6		7.6	7.3
399	Bolus	1.7			1.5		1.5		1.4	1.5
436	Bolus	0.5			0.5		0.6		0.5	0.6
458	Bolus	0.2			0.2		0.2		0.2	0.2
519	Bolus	0.3			0.3		0.3		0.3	0.3
528	Bolus	3.5			4.0		4.3		4.6	4.9
529	Bolus	0.5			0.4		0.4		0.4	0.4
102	Bolus + Vit C	4.8			5.0		5.1		5.1	5.5
209	Bolus + Vit C	0.5			0.5		0.5		0.5	0.5
258	Bolus + Vit C	6.8			7.0		7.8		7.6	7.8
299	Bolus + Vit C	3.0			2.8		3.0		2.9	2.9
333	Bolus + Vit C	13.2			12.4		11.0		11.9	12.1
341	Bolus + Vit C	1.9			1.7		1.7		1.6	1.7
354	Bolus + Vit C	0.4			0.4		0.3		0.4	0.3
390	Bolus + Vit C	0.3			0.2		0.2		0.3	0.2
393	Bolus + Vit C	0.3			0.2		0.2		0.2	0.2
396	Bolus + Vit C	6.4			6.3		1.3		6.3	6.0
399	Bolus + Vit C	1.0			1.0		1.0		0.9	1.0
436	Bolus + Vit C	3.4			3.2		3.4		3.5	3.6
458	Bolus + Vit C	0.2			0.2		0.2		0.2	0.2
519	Bolus + Vit C	0.3			0.3		0.3		0.3	0.3
528	Bolus + Vit C	3.4			3.2		3.0		2.9	2.9
529	Bolus + Vit C	0.5			0.4		0.3		0.3	0.3
102	Sipping	1.2			1.1		6.1		1.2	1.2
209	Sipping	0.7			0.7		0.7		0.8	0.7
258	Sipping	6.1			5.8		5.8		6.1	6.0
299	Sipping	3.6			3.7		3.6		3.8	3.9
333	Sipping	11.8			11.0		11.3		11.5	11.0
341	Sipping	0.6			0.6		0.5		0.5	0.6
354	Sipping	0.6			0.6		0.5		0.5	0.5
390	Sipping	0.4			0.4		0.3		0.4	0.3
393	Sipping	0.3			0.2		0.2		0.2	0.2
396	Sipping	5.8			5.8		6.0		6.2	6.2
399	Sipping	1.2			1.0		1.0		1.0	1.0
436	Sipping	0.7			0.6		0.5		0.6	0.5
458	Sipping	0.2			0.3		0.2		0.2	0.2
519	Sipping	0.5			0.5		0.4		0.5	0.5
528	Sipping	3.2			3.2		3.5		2.8	4.2
529	Sipping	0.3			0.3		0.2		0.3	0.3
102	Sipping + Vit C	1.5			1.5		1.5		1.5	1.4
209	Sipping + Vit C	0.5			0.5		0.5		0.5	0.5
258	Sipping + Vit C	4.9			4.9		4.7		4.6	4.7
299	Sipping + Vit C	4.1			3.8		1.7		4.0	1.6
333	Sipping + Vit C	15.8			15.3		14.6		15.3	15.5
341	Sipping + Vit C	0.5			0.6		0.6		0.6	0.6
354	Sipping + Vit C	0.5			0.5		0.4		0.4	0.4
390	Sipping + Vit C	0.4			0.3		0.3		0.3	0.3
393	Sipping + Vit C	0.2			0.2		0.2		0.2	0.2
396	Sipping + Vit C	5.1			4.8		4.6		3.9	4.2
399	Sipping + Vit C	1.6			1.7		1.8		1.7	1.9
436	Sipping + Vit C	0.2			0.9		0.9		0.8	0.7
458	Sipping + Vit C	0.2			0.2		0.2		0.2	0.2
519	Sipping + Vit C	1.7			1.5		1.4		1.4	1.4
528	Sipping + Vit C	4.0			3.7		3.7		3.5	3.3
529	Sipping + Vit C	0.4			0.3		0.3		0.3	0.3

Supplementary Material:

Raw Data: Conjugated Dienes (μmol/L)

ID	Treatment	0min	30min	60min	120min	180min	240min	270min	300min	360min
102	Bolus	50.5			65.3	37.0	61.0		69.7	67.2
209	Bolus	55.5			39.5	40.7	37.0		43.8	38.8
258	Bolus	39.5			41.9	40.1	40.7		11.7	47.5
299	Bolus	59.8			66.0	65.3	48.1		51.2	72.1
333	Bolus	44.4			60.4	63.5	45.0		41.9	61.0
341	Bolus	63.5			60.4	30.8	46.8		64.7	52.4
354	Bolus	38.2			56.7	5.6	46.2		17.3	32.1
390	Bolus	64.7			30.8	27.7	39.5		31.4	50.5
393	Bolus	47.5			41.3	70.3	61.6		54.2	56.7
396	Bolus	76.4			53.0	27.7	52.4		43.8	47.5
399	Bolus	31.4			45.0	30.8	86.3		119.0	40.7
436	Bolus	33.3			29.6	26.5	41.9		53.6	24.7
458	Bolus	53.6			33.3	40.7	22.8		42.5	37.0
519	Bolus	35.1			40.7	23.4	30.2		20.3	38.8
528	Bolus	16.6			17.3	18.5	16.0		13.6	19.1
529	Bolus	35.8			38.2	48.7	36.4		29.0	46.8
102	Bolus + Vit C	53.6			69.7	59.2	59.2		61.6	65.3
209	Bolus + Vit C	43.8			42.5	43.1	41.3		38.8	41.9
258	Bolus + Vit C	56.7			34.5	37.6	70.9		38.2	33.9
299	Bolus + Vit C	42.5			66.6	35.1	43.8		38.2	59.2
333	Bolus + Vit C	56.7			97.4	41.3	72.1		32.7	66.6
341	Bolus + Vit C	53.6			59.8	35.8	42.5		72.1	45.0
354	Bolus + Vit C	12.3			22.2	18.5	40.1		27.1	29.0
390	Bolus + Vit C	42.5			37.0	66.0	46.8		43.8	51.2
393	Bolus + Vit C	72.1			46.8	17.9	54.2		64.1	54.2
396	Bolus + Vit C	38.2			51.2	40.7	38.8		35.8	52.4
399	Bolus + Vit C	66.0			43.1	30.8	33.9		40.7	62.9
436	Bolus + Vit C	56.1			21.0	24.0	27.1		22.2	27.7
458	Bolus + Vit C	51.8			35.8	32.1	13.6		45.6	40.7
519	Bolus + Vit C	42.5			48.1	32.7	37.6		55.5	29.0
528	Bolus + Vit C	8.6			27.1	19.1	23.4		11.1	21.0
529	Bolus + Vit C	29.6			28.4	31.4	35.1		30.8	36.4
102	Sipping	51.2			82.6	73.3	67.8		88.8	110.9
209	Sipping	37.6			33.3	39.5	46.2		42.5	48.1
258	Sipping	69.7			38.8	43.8	38.2		40.7	29.6
299	Sipping	74.0			62.3	43.8	35.1		69.0	46.2
333	Sipping	57.3			29.6	43.8	117.1		19.7	12.9
341	Sipping	48.1			47.5	48.7	47.5		41.9	54.9
354	Sipping	64.1			34.5	11.1	6.8		26.5	40.7
390	Sipping	46.2			37.0	42.5	39.5		53.6	54.9
393	Sipping	65.3			59.2	63.5	49.3		122.0	69.7
396	Sipping	27.7			22.2	45.0	36.4		61.0	40.7
399	Sipping	48.7			61.6	52.4	74.0		50.5	80.7
436	Sipping	28.4			29.0	19.7	37.6		30.8	96.2
458	Sipping	24.0			30.8	27.1	37.6		29.6	45.6
519	Sipping	66.0			59.2	60.4	51.8		59.2	59.2
528	Sipping	14.8			12.9	27.7	8.0		11.7	16.0
529	Sipping	40.7			32.7	36.4	47.5		40.1	34.5
102	Sipping + Vit C	56.7			111.6	56.1	57.9		70.3	117.1
209	Sipping + Vit C	81.4			54.2	34.5	49.3		33.9	59.8
258	Sipping + Vit C	75.2			40.7	32.1	35.1		35.1	32.7
299	Sipping + Vit C	46.8			38.2	54.2	60.4		30.8	46.2
333	Sipping + Vit C	33.9			41.3	45.6	43.1		32.7	63.5
341	Sipping + Vit C	46.8			61.0	32.1	38.2		49.9	54.2
354	Sipping + Vit C	60.4			15.4	48.7	34.5		26.5	33.3
390	Sipping + Vit C	64.7			53.0	46.2	68.4		82.0	58.6
393	Sipping + Vit C	62.3			51.8	62.3	54.9		80.7	75.2
396	Sipping + Vit C	80.1			37.0	51.8	48.7		32.1	43.1
399	Sipping + Vit C	64.1			18.5	61.0	39.5		52.4	45.0
436	Sipping + Vit C	26.5			27.7	31.4	24.0		30.2	31.4
458	Sipping + Vit C	32.1			108.5	28.4	30.8		65.3	28.4
519	Sipping + Vit C	35.1			29.6	41.9	45.0		41.3	41.9
528	Sipping + Vit C	6.2			27.1	13.6	19.1		16.0	12.9
529	Sipping + Vit C	66.0			25.9	27.7	25.3		31.4	33.3

Supplementary Material:

Raw Data: ApoB-100 (µmol/L)

ID	Treatment	0min	30min	60min	120min	180min	240min	270min	300min	360min
102	Bolus	3.14			3.16	2.84	3.34		3.42	2.53
209	Bolus	3.53			3.24	2.94	2.83		3.38	3.01
258	Bolus	2.86			2.18	2.54	2.41		2.66	2.92
299	Bolus	2.52			3.40	3.52	4.04		1.87	2.70
333	Bolus	2.47			1.75	2.70	2.76		2.64	2.67
341	Bolus	2.49			2.97	2.94	2.58		2.94	2.56
354	Bolus	2.56			2.64	2.63	2.61		3.18	2.59
390	Bolus	2.64			2.66	2.76	2.68		2.62	2.73
393	Bolus	2.79			2.43	2.68	2.79		2.91	2.83
396	Bolus	2.76			2.67	2.81	2.55		2.89	2.89
399	Bolus	2.37			1.58	2.56	2.70		2.35	2.60
436	Bolus	2.80			3.15	3.07	2.62		2.43	2.64
458	Bolus	3.59			2.87	3.81	3.09		2.62	3.00
519	Bolus	2.82			2.54	2.70	3.00		2.53	2.81
528	Bolus	1.99			2.50	2.99	2.82		2.31	2.69
529	Bolus	2.70			3.30	2.68	2.78		3.61	2.69
102	Bolus + Vit C	2.68			2.62	3.81	3.76		2.99	3.62
209	Bolus + Vit C	2.78			3.42	2.71	2.90		2.57	2.68
258	Bolus + Vit C	2.86			3.14	2.87	2.55		2.88	2.80
299	Bolus + Vit C	3.81			3.46	3.71	3.03		3.54	2.78
333	Bolus + Vit C	2.88			2.70	2.54	2.70		2.64	2.71
341	Bolus + Vit C	3.03			3.03	2.80	2.59		2.65	3.06
354	Bolus + Vit C	2.69			2.58	2.64	2.81		2.55	2.60
390	Bolus + Vit C	2.43			2.58	2.69	2.71		2.77	2.63
393	Bolus + Vit C	2.90			2.35	2.47	2.82		3.02	2.92
396	Bolus + Vit C	2.88			3.41	2.54	3.03		2.64	3.02
399	Bolus + Vit C	2.70			2.52	2.55	2.66		2.70	2.64
436	Bolus + Vit C	2.67			2.55	2.50	2.93		2.76	2.92
458	Bolus + Vit C	2.51			2.96	2.77	3.08		2.74	3.01
519	Bolus + Vit C	3.10			2.93	3.03	3.12		2.85	2.92
528	Bolus + Vit C	2.88			2.90	2.87	2.81		2.73	3.14
529	Bolus + Vit C	2.68			3.20	2.66	2.84		3.07	2.80
102	Sipping	3.62			2.72	3.45	3.35		3.58	3.24
209	Sipping	2.36			2.50	2.90	3.05		3.00	2.84
258	Sipping	3.62			2.92	2.85	2.85		3.39	2.96
299	Sipping	3.41			3.77	3.42	3.67		3.57	3.57
333	Sipping	2.51			2.70	2.60	2.70		2.70	2.69
341	Sipping	3.02			2.71	3.16	2.72		3.17	3.09
354	Sipping	2.57			3.03	2.77	3.08		2.54	2.55
390	Sipping	2.80			2.95	2.79	2.65		3.09	2.59
393	Sipping	3.42			2.23	2.17	2.19		2.51	2.55
396	Sipping	2.95			3.34	2.44	2.69		3.01	3.51
399	Sipping	2.70			2.70	2.70	2.76		2.70	2.70
436	Sipping	2.86			2.92	2.82	2.81		3.10	2.84
458	Sipping	2.37			2.52	2.89	2.31		2.60	2.63
519	Sipping	2.73			2.68	2.73	2.48		3.23	2.70
528	Sipping	3.22			2.76	3.19	2.61		2.70	2.50
529	Sipping	2.85			2.68	3.33	2.82		3.00	3.30
102	Sipping + Vit C	2.57			2.73	3.33	2.93		3.16	2.64
209	Sipping + Vit C	3.02			2.98	2.69	2.87		2.82	3.06
258	Sipping + Vit C	3.24			2.75	3.00	3.12		2.44	3.11
299	Sipping + Vit C	3.64			3.56	3.15	3.19		3.83	2.98
333	Sipping + Vit C	3.03			2.92	2.50	2.70		2.61	2.71
341	Sipping + Vit C	2.63			3.17	2.68	2.89		2.71	3.02
354	Sipping + Vit C	2.82			2.59	3.30	2.70		2.96	3.01
390	Sipping + Vit C	2.82			2.74	2.49	2.92		2.65	2.59
393	Sipping + Vit C	2.05			2.55	2.30	2.61		3.08	2.92
396	Sipping + Vit C	2.58			2.57	2.69	2.62		2.83	2.88
399	Sipping + Vit C	2.80			2.66	2.76	2.70		2.70	2.62
436	Sipping + Vit C	2.76			3.09	2.48	2.95		2.71	2.64
458	Sipping + Vit C	2.58			2.90	2.73	2.82		2.60	2.63
519	Sipping + Vit C	3.20			3.49	3.47	5.05		5.04	6.76
528	Sipping + Vit C	2.91			3.04	2.68	2.74		2.97	3.19
529	Sipping + Vit C	3.07			2.83	2.62	3.53		3.05	3.36
